# Human papillomavirus (HPV) prediction for oropharyngeal cancer based on CT by using off‐the‐shelf features: A dual‐dataset study

**DOI:** 10.1002/acm2.70061

**Published:** 2025-03-02

**Authors:** Junhua Chen, Yanyan Cheng, Lijun Chen, Banghua Yang

**Affiliations:** ^1^ School of Medicine Shanghai University Shanghai China; ^2^ Medical Engineering Department Shandong Provincial Hospital Affiliated to Shandong First Medical University Shandong China; ^3^ The Fourth People's Hospital of Jiangshan City Quzhou China; ^4^ School of Mechatronic Engineering and Automation, Research Center of Brain Computer Engineering Shanghai University Shanghai China

**Keywords:** 3D deep features, image‐based HPV prediction for oropharyngeal cancer, Siamese neural network, transfer learning

## Abstract

**Background:**

This study aims to develop a novel predictive model for determining human papillomavirus (HPV) presence in oropharyngeal cancer using computed tomography (CT). Current image‐based HPV prediction methods are hindered by high computational demands or suboptimal performance.

**Methods:**

To address these issues, we propose a methodology that employs a Siamese Neural Network architecture, integrating multi‐modality off‐the‐shelf features—handcrafted features and 3D deep features—to enhance the representation of information. We assessed the incremental benefit of combining 3D deep features from various networks and introduced manufacturer normalization. Our method was also designed for computational efficiency, utilizing transfer learning and allowing for model execution on a single‐CPU platform. A substantial dataset comprising 1453 valid samples was used as internal validation, a separate independent dataset for external validation.

**Results:**

Our proposed model achieved superior performance compared to other methods, with an average area under the receiver operating characteristic curve (AUC) of 0.791 [95% (confidence interval, CI), 0.781–0.809], an average recall of 0.827 [95% CI, 0.798–0.858], and an average accuracy of 0.741 [95% CI, 0.730–0.752], indicating promise for clinical application. In the external validation, proposed method attained an AUC of 0.581 [95% CI, 0.560–0.603] and same network architecture with pure deep features achieved an AUC of 0.700 [95% CI, 0.682–0.717]. An ablation study confirmed the effectiveness of incorporating manufacturer normalization and the synergistic effect of combining different feature sets.

**Conclusion:**

Overall, our proposed model not only outperforms existing counterparts for HPV status prediction but is also computationally accessible for use on a single‐CPU platform, which reduces resource requirements and enhances clinical usability.

## 1 INTRODUCTION

In 2020, an estimated 476 125 individuals globally were diagnosed with oral or oropharyngeal cancer, resulting in approximately 225 900 deaths attributed to these conditions.^1^ Infection with the human papillomavirus (HPV), especially type 16, has been identified as a significant risk factor for oropharyngeal cancers, notably affecting the tonsils or the base of the tongue.^2^ Furthermore, HPV presence serves as an important prognostic biomarker for treatment outcomes.^3^ In clinical settings, the predominant methods for detecting HPV include DNA polymerase chain reaction (PCR), p16 immunohistochemistry (IHC), DNA/RNA in situ hybridization,^4^ etc. Notably, p16 immunohistochemistry, the most commonly utilized method, has achieved a sensitivity of over 90% and a specificity of over 80%.^5^ However, PCR and IHC are generally time‐consuming and, in some instances, invasive tests for patients with oropharyngeal cancer. This article proposes a novel predictive methodology for HPV detection in oropharyngeal cancer using CT imaging techniques.

One major limitation of existing traditional image‐based methods for predicting HPV presence is the potential influence of coincidental feature selection and dataset bias on their high performance, with many studies drawing conclusions from relatively small datasets. Additionally, state‐of‐the‐art (SOTA) deep learning‐based methods for HPV prediction face challenges, including high computational demands and limited accessibility to pre‐trained models, diminishing their impact among critical stakeholders such as clinical researchers and medical physicists.^6^

Image‐based HPV prediction is a challenging task in medical image analysis domain, and from the perspective of methodology, major methods adopt machine learning methods to solve this question.^6^ These prediction algorithms can be broadly categorized based on the nature of feature extraction into two types: methods based on hand‐crafted features and those utilizing deep learning approaches.^7^ We will briefly review literatures started from hand‐craft features based methods to deep features‐based methods.

Historically, methods based on hand‐crafted features dominated the field of HPV prediction, with Radiomics being a typical example of such features.^8^ In these studies. a small subset of features are chosen for HPV prediction, often referred to as a “signature” in the literature.^9^, ^10^ Classical approaches adhering to this pipeline have been published, random forest achieving an AUC of 0.73.^11^ While hand‐crafted feature‐based methods have demonstrated promising results, however, there are some limitations. Specifically, the potential influence of coincidental feature selection and dataset bias on their high performance cannot be overlooked. Additionally, many studies report findings based on small datasets, which may compromise the generalizability of the proposed methods to other datasets.^12^

In the era of deep learning, novel approaches have emerged for image‐based HPV prediction. Deep learning methods, leveraging deep features as an alternative to radiomics,^13^ utilize a variety of pre‐trained networks. Deep learning based methods for HPV prediction have demonstrated promising results, achieving an AUC of 0.83 in internal validation and 0.88 in external validation highlighting their potential utility in clinical practice.^14^ A major drawback of STOA methods^14^ is that they are not the off‐the‐shelf features based studies and their heavy dependence on graphics processing units (GPUs) for model training creates accessibility challenges for key users, such as clinical researchers and medical physicists.^15^

Regarding imaging modalities for image‐based HPV prediction, magnetic resonance imaging (MRI), computed tomography (CT), and ultrasound are the primary options^6^, ^14^ The high accessibility and affordability of ultrasound have facilitated its use in head and neck examinations and image‐based HPV prediction.^16^ However, its low signal‐to‐noise ratio and resolution limit its application in complex image analysis tasks. MRI provides a wider range of soft tissue contrast and demonstrates greater sensitivity and specificity in identifying head and neck abnormalities.^17^ MRI‐based HPV prediction models have achieved only moderate performance due to the smaller dataset sizes, attributed to MRI's longer acquisition times and limited accessibility in‐developing regions.^18^ CT imaging, favored in clinical practice for oropharyngeal cancer due to its quicker imaging process and greater accessibility, has shown better performance in HPV prediction tasks, with STOA CT‐based algorithms achieving an AUC of 0.83.^13^, ^19^ This study proposes the development of an HPV prediction model using CT images, aiming for a balance between accessibility, non‐invasiveness, and predictive accuracy.

Our aim is to develop a predictive model that does not require high‐performance GPU training and incorporates hybrid off‐the‐shelf features—radiomics and 3D deep features—to enhance the representational information of features, majority reasons for extracting features in off‐the‐shelf manner is reducing the computation for building model. For CT images, 3D deep features were extracted from the output layer of action recognition networks pre‐trained on natural videos. The rationale for this approach detailed in previously published study,^20^ a summary will be provided in the discussion section. To effectively integrate features from diverse sources, we employed a Siamese neural network (SNN) as the backbone of our classifier; the network inputs for our study include radiomics and deep features extracted from various video action recognition artificial neural networks. Additionally, we examined the marginal effect of combining deep features from various established action recognition networks.

Major contributions of this study are as follows:

1. The development of a pure off‐the‐shelf features‐based HPV prediction model, which surpasses its counterparts in performance.
2. In order to bolster the reliability of our findings, this study was conducted using a large dataset comprising 1453 valid samples. Furthermore, external validation was performed in alignment with the Transparent Reporting for Individual Prognosis Or Diagnosis statement, categorizing our study as a type 3a study.^21^
3. Through ablation studies, this study identifies a novel phenomenon: deep feature based classifiers outperform hand‐crafted feature‐based classifiers during external validation.
4. All model training and validation on a single‐CPU platform, which reduces resource requirements and enhances clinical usability.

In summary, the main novelty of this study lies in proposing an image‐based HPV prediction method for oropharyngeal cancer using off‐the‐shelf features. This approach achieves competitive performance while requiring fewer computational resources, thereby enhancing its potential clinical applicability.

Fanizzi et al.^22^ proposed a novel CT‐based method for predicting HPV status in oropharyngeal cancer. They employed a CNN‐based model and reported an AUC of 0.73 and an accuracy of 0.65 on an independent external test set, with high interpretability. The key differences between their study and the current research lie in the approach to deep feature extraction. Our study exclusively utilized pure transfer learning, while Fanizzi et al.’s method extracted deep features combined with GPU‐accelerated training.

To facilitate transparency and reproducibility, we are making the source code of our study publicly available. The source code, alongside the Radiomics and deep features, data for statistical analysis, and supporting materials, can be accessed at our repository.^23^

## 2 METHODS

**FIGURE 1:**
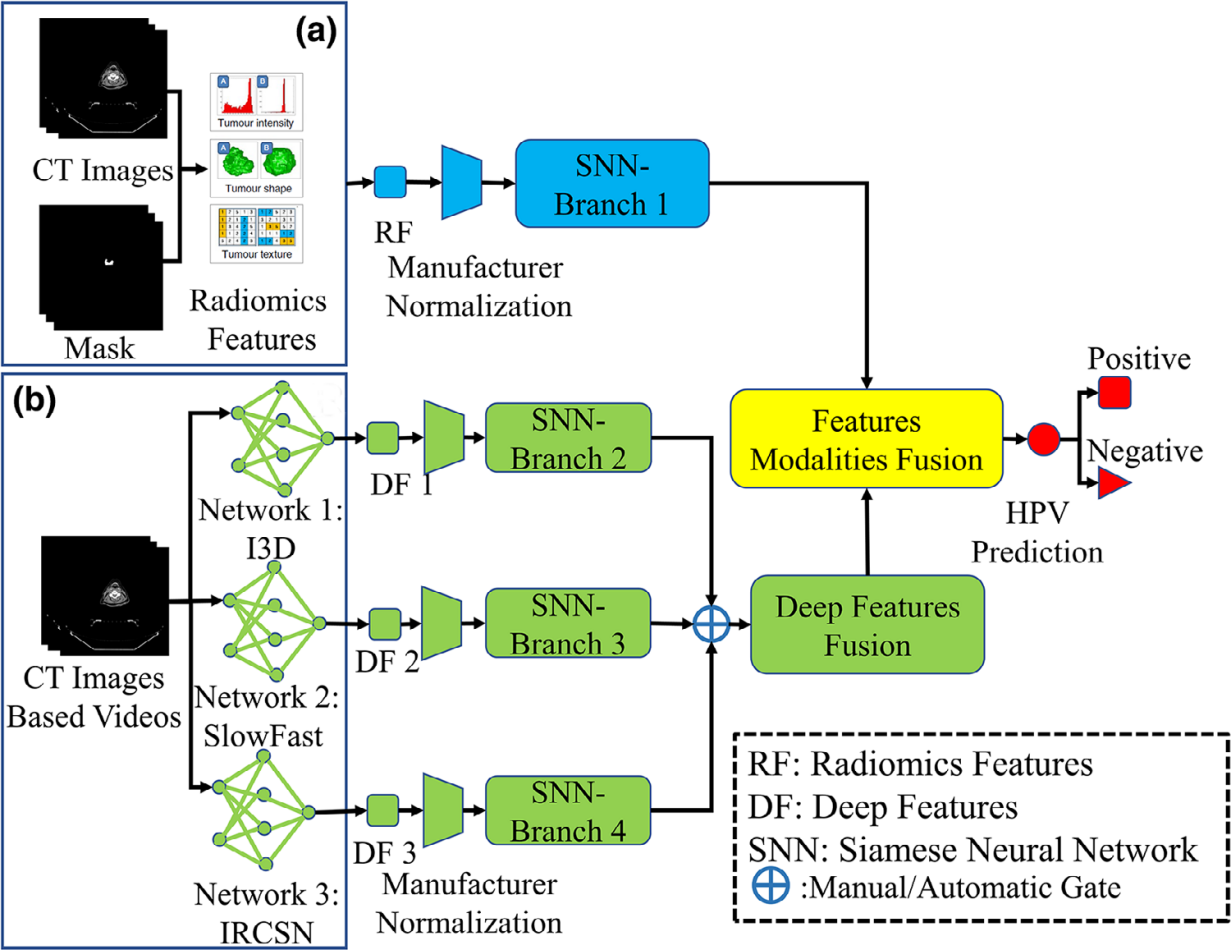
Pipeline of study. (a) Radiomics features and its related analysis workflow; (b) Deep features and its related analysis workflow. DF, deep features; I3D, Inflated 3D ConvNet; IRCSN, channel‐separated convolutional networks; RF, radiomics features; Slowfast, Slowfast network; SNN, Siamese neural network.

Institutional review board (IRB) approval was deemed unnecessary for this study due to the utilization of an open‐access data collection from The Cancer Imaging Archive, where all patient‐specific private information had been anonymized in the CT scans.^24^ The methodology of our study is delineated in Figure 1.

### 2.1 Data acquisition

As delineated in the introductory section, this research leverages a substantial dataset, the CT images from the Large Head and Neck Cohort (RADCURE), for model training and validation, in addition to a comparatively smaller dataset, HEAD‐NECK‐RADIOMICS (HN1) for external validation of the model. External validation in our study means evaluating the HPV detection model's performance on a test dataset not used during the “internal validation,” which employed cross validation. A succinct overview of these datasets is provided below.

The RADCURE dataset encompasses information on 3346 patients diagnosed with oropharyngeal cancer, including CT images along with delineations of normal and abnormal tissue contours. The tumor HPV status was ascertained using IHC and/or HPV DNA PCR, with test results available for 1717 patients within the RADCURE dataset. RTSTUCT files was absent for some patients and finally 1453 samples available for study in following analysis. All available samples were incorporated into subsequent analyses. The RADCURE dataset samples was used for the training and validation of the HPV prediction model, and a detailed index of patients eligible for inclusion in the RADCURE dataset is available in Table S1 in the Supporting Information as mentioned above.

H&N1 dataset encompasses data from 137 patients with head and neck squamous cell carcinoma, including CT images along with delineations of normal and pathological tissue contours. The dataset predominantly includes 88 cases of oropharyngeal cancer, excluding larynx cancer, which have been considered for subsequent analyses. The tumor HPV status was assessed using PCR, with results available for 81 patients in the H&N1 dataset. The H&N1 dataset samples were employed for the external validation of the HPV prediction model, and a detailed index of the patients eligible for inclusion is presented in Table S2 in the Supporting Information. Notably, retaining data for this external validation was deemed unnecessary.

**FIGURE 2:**
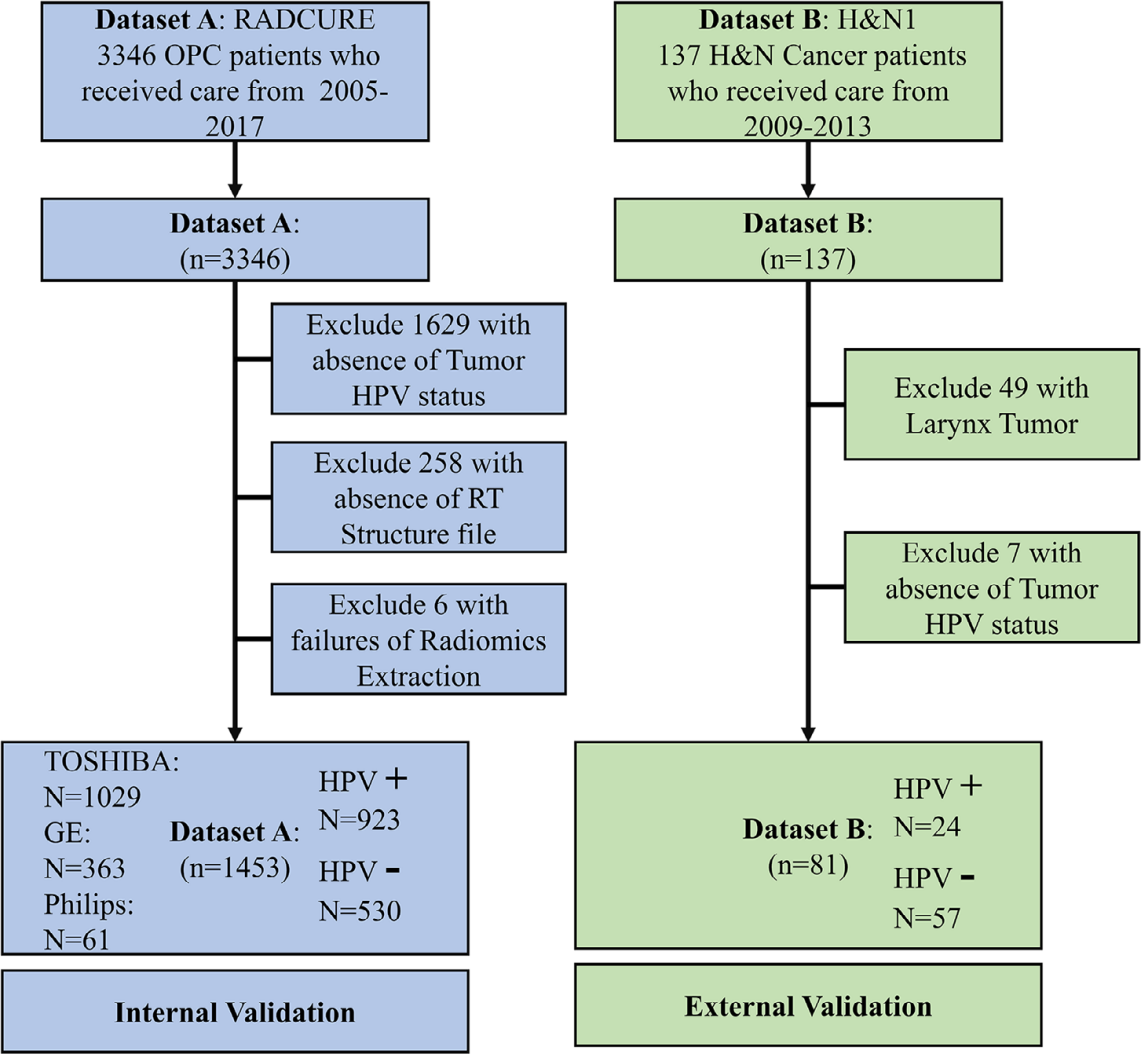
Flowchart shows samples exclusion for two datasets. Sample exclusion criteria for two datasets including absence of tumor HPV status or RT structure file, failure in radiomics feature extraction, and divergence in tumor phenotype. RT, radiotherapy.

The exclusion criteria for samples in both datasets are depicted in the flowchart presented in Figure 2. Statistical analysis of HPV status for both datasets and scanner vendors for RADCURE dataset are available in Figure 2 as well.

### 2.2 Radiomics features extraction

For the extraction of radiomics features, delineating the region of interest (ROI) was imperative, particularly the tumor contours in the RADCURE dataset, which were archived in RTSTRUCT files.^25^ We generated a 3D mask of the tumor employing custom scripts, facilitated by the RT‐Utils package focusing on the contour designated as GTVp (primary gross tumor volume) for mask reconstruction.^26^ The H&N1 dataset inherently includes the original 3D tumor mask files.

In terms of radiomics feature extraction procedures, we utilized the open‐source Python library, pyRadiomics (version 2.2.0), to extract a total of 103 radiomics features. For comprehensive details on the extracted features, refer to Table S3 in the Supporting Information. The human interpretability of radiomics features refers to how easily clinicians and researchers can understand and relate these features to clinical or biological information. Improving interpretability involves correlating features with clinical outcomes and integrating expert insights to provide context.^27^ The specific configurations employed for feature extraction with pyRadiomics are documented in Table S4 in the Supporting Information.

### 2.3 Deep features extraction

The standardization of radiomics, a focal point of research over the past decade, has been extensively explored.^28^ Consequently, radiomics features were extracted a single time utilizing a conventional parameter setting (detailed in Table S4 in the Supporting Information). A similar standardization issue has also been raised in studies on deep features,^29^ where reproducibility is influenced by various factors, including pre‐trained datasets, image pre‐processing, network architectures, output layers, convolutional filters, etc. To minimize the impact of these factors on deep feature reproducibility—excluding network architectures—we extracted deep features from the output layers of pre‐trained action recognition 3D neural networks using the “Kinetics 400 dataset.” “Kinetics 400 dataset,” a large‐scale, high‐quality video dataset comprises 300 000 video clips of 400 human action classes for action recognition research.

On the other hand, the independence and synergistic effects of 3D features derived from various action recognition networks remain uncertain, particularly in applications related to medical imaging. Inspired by the AdaBoost algorithm,^30^, ^31^ which demonstrates that a robust classifier can emerge from the combination of several weaker classifiers, we hypothesized that classifiers based on deep features from a single pre‐trained action recognition network could act as a weak classifier. A more potent classifier could be developed by amalgamating several deep feature‐based classifiers. Hence, the investigation into the marginal effects of integrating deep features from different networks into a single classifier presents a novel inquiry for this study.

As depicted in Figure 1, we extracted deep features directly from three distinct networks—Inflated 3D ConvNet (I3D),^32^ SlowFast Networks,^33^ and IRCSN Networks^34^—all of which were pre‐trained on the same dataset. This was accomplished with the assistance of the GluonCV deep learning platform.^35^ The specific pre‐trained models from the GluonCV platform for these networks are “i3d_nl10_resnet101_v1_kinetics400”, “slowfast_4 × 16_resnet50_kinetics400” and “r2plus1d_v2_resnet152_kinetics400,” respectively. A manual gate was integrated into the network architecture to regulate the utilization of the deep feature branch (as shown in Figure 1), allowing for seven potential deep feature branch combinations for our classifier.

For the extraction of deep features from CT images, we converted the images into video format, with each slice representing a frame, using custom scripts. The windowing of CT images significantly impacts their visual representation and was thus standardized prior to video generation, setting the window level and width for CT scans in the RADCURE and H&N1 datasets at 40 and 300 HU, respectively. Additionally, the tumor masks used for calculating radiomics features were substituted with bounding boxes, standardized to a size of 128 × 128 pixel, to facilitate the extraction of deep features from the ROI. Ultimately, 400 deep features were extracted from the three networks, with the specific attributes for action recognition of each deep feature task detailed in Table S5 in the Supporting Information.

### 2.4 Manufacturer bias normalization

The RADCURE dataset, originating from a single center and adhering to a similar image acquisition protocol, presents limited disturbance in texture and related features attributable to the acquisition protocol itself. However, a deeper analysis revealed variability in the imaging equipment used across the dataset, particularly noted within the RADCURE collection. Specifically, 363 samples were acquired using GE scanners, 1029 samples from TOSHIBA scanners, and the remaining 61 samples from Philips scanners. The variation in scanner brands introduces a notable factor affecting the repeatability and reproducibility of features, which in turn impacts the performance of features‐based computer‐aided diagnosis system.^36^ Consequently, it is imperative to mitigate the influence of scanner bias on predictions.

In alignment with methodologies from purely radiomic feature‐based studies,^37^ we employed the ComBat algorithm—an algorithm with a goal to harmonize batch effects in data to ensure consistency and comparability across different datasets—to minimize the impact of vendor bias across radiomics features, deep features, and their associated prediction models, a process we refer to as manufacturer bias normalization.^38^ During the implementation of our manufacturer bias normalization, ComBat harmonization (without the empirical Bayes assumption, using parametric adjustments, and incorporating three batches) was applied to all radiomic and deep features. Features extracted from the majority manufacturer, Toshiba, were referenced as the “gold standard” for normalization.

### 2.5 Classifier building

**FIGURE 3:**
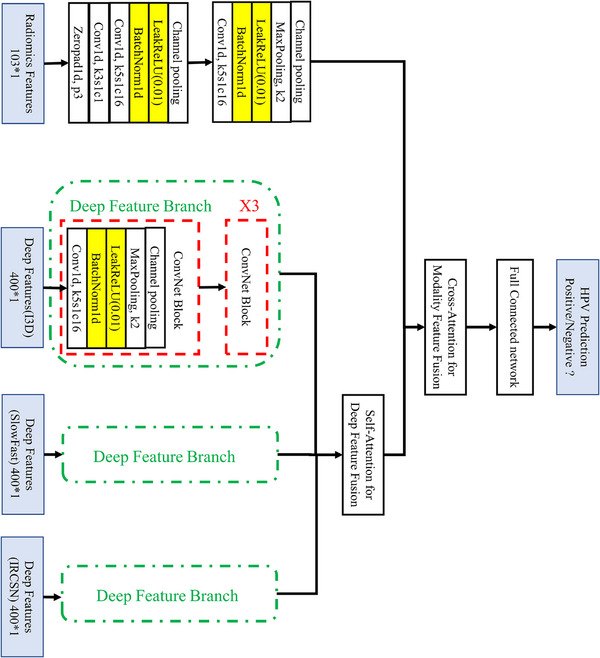
Architecture of prediction model for HPV status. 103*1 means the dimension of radiomics features; 400*1 means the dimension of deep features. X3 means triple ConvNet Blocks.

The network architecture designed for HPV prediction is detailed in Figure 3 and basic neural network of SNN's each branch is 1D conventional neural networks. As shown in Figure 3, deep features from various networks were integrated using a self‐attention mechanism, and then followed by another cross‐attention mechanism‐based feature fusion layer for both deep features and radiomics. We adopted cross entropy as the cost function for our classifier, with stochastic gradient descent chosen as the optimization algorithm for training the network.

### 2.6 Experiments and statistical analysis

The construction and validation of the classifier were conducted using Python 3.6, GluonCV 0.8, and TensorFlow 2.6.0 on a Core i5‐13600KF CPU equipped with 32GB of RAM. For this phase of the study, 1453 samples from the RADCURE dataset were selected. The methodology involved 20 iterations of complete training and validation cycles, analyzing radiomics and deep features through five‐fold cross‐validation in each iteration. To mitigate the risk of overfitting, an early stopping strategy was employed. Consequently, the neural network was trained over 200 epochs, with each iteration lasting approximately 8–9 min. The initial learning rate was established at 0.0001, decreasing by a factor of 0.8 every 50 epochs and batch size for model training was set as 1.

For the model's external validation, 81 samples from the H&N 1 dataset were utilized. This phase adhered to the same procedural framework as the internal validation based on the RADCURE dataset, in other words, that five‐fold cross‐validation was performed on the external dataset too. Performance metrics for both internal and external validations included recall, accuracy, and the AUC.

To thoroughly assess the efficacy of the proposed method, it was benchmarked against several existing off‐the‐shelf features‐based studies^13^, ^39^—one hand‐crafted based classifier and one deep feature‐based classifier. This investigation specifically focused on applications leveraging pure transfer learning, thereby excluding studies that centered on model development utilizing GPU‐accelerated training.

To explore the marginal effect of integrating deep features from multiple networks, this study included a comparison of prediction models varying in the number of branches of deep features as part of an ablation study. Additionally, this research investigated the performance of the model across different manufacturers to demonstrate the impact of scanner bias and the necessity of manufactures normalization. Due to limited valid samples collected from Philips scanners, scanner bias ablation studies for Philips will be absent. Furthermore, to assess the benefits of incorporating manufacturer bias normalization into the prediction model, a corresponding ablation study was conducted. This comprehensive analysis aims to elucidate the potential advantages of these methodological enhancements in improving the accuracy and generalizability of the prediction model.

## 3 RESULTS

### 3.1 HPV prediction results in the RADCURE dataset

**FIGURE 4:**
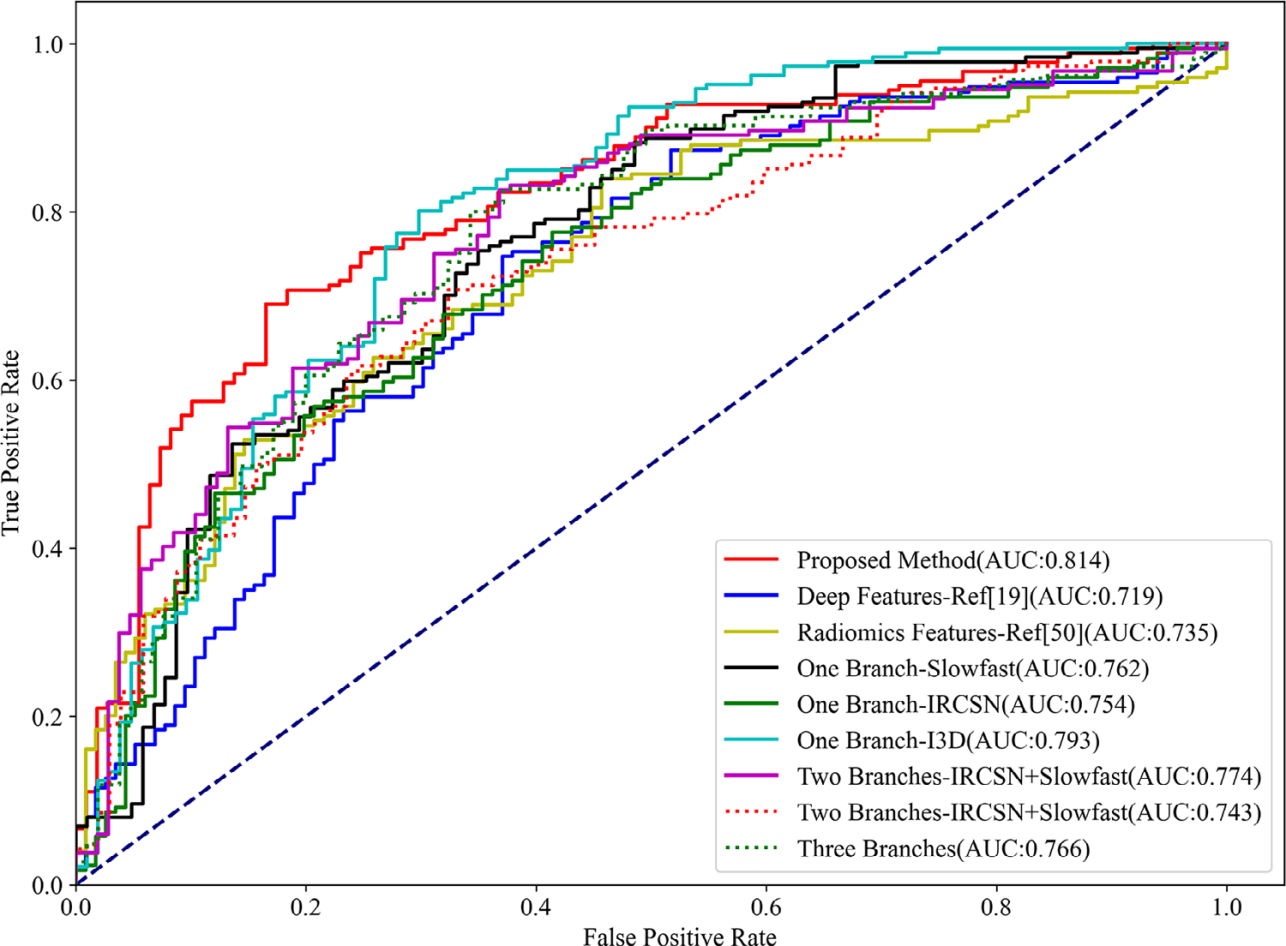
An example of AUC curves of HPV prediction for different methods with same training and testing data. The diagonal represents the performance of a random classifier, different methods refers methods list in Table 1.

Results of built prediction model for HPV status in RADCURE dataset based on 20 iterations of five‐fold cross‐validation are shown in Table 1. Figure 4 presents a representative set of AUC curves for the different methods, the same training and testing data (80% of the samples for training and 20% for testing) were used in this representative experiment for all methods. The results shown network achieved best performance when radiomics features and deep features from Inflated 3D Conv Net, Slow Fast Networks available for classifier. The best architecture of proposed classifier (I3D + SlowFast) achieved an AUC of 0.791 (95% confidence intervals (CI), [0.781–0.809]), an average Recall of 0.827 (95% CI, [0.798–0.858]), and an average accuracy of 0.741 (95% CI, [0.730–0.752]). Proposed method achieved best result in AUC and Accuracy metric compared with other methods and acceptable result in recall metric (not worse than other methods statistically, Wilcoxon rank‐sum test). For context, a previous study referenced in the Introduction, which utilized CT deep features from a highly GPU‐intensive network, reported an AUC of 0.83, albeit on different datasets.

**TABLE 1:** Prediction results of HPV status for oropharyngeal cancer in RADCURE dataset.

Methods/Metrics	AUC	Recall	Accuracy
Proposed method (I3D+ SlowFast)	0.791 [0.781, 0.809]^a^	0.827 [0.798, 0.858]	0.741 [0.730, 0.752]
Hand‐crafted features‐based method^29^	0.748 [0.734,0.761] p≪0.01 ^*^	0.847 [0.832, 0.863] p=0.50	0.708 [0.695,0.721] p<0.01
Deep features (DF) based method (I3D)^13^	0.713 [0.698, 0.728] p≪0.01	0.858 [0.829, 0.888] p=0.15	0.685 [0.673,0.697] p≪0.01
One DF branch method (I3D)	0.772 [0.759, 0.786] p=0.04	0.834 [0.814, 0.855] p=0.99	0.727 [0.712, 0.743] p=0.13
One DF branch method (SlowFast)	0.777 [0.761, 0.792] p=0.17	0.823 [0.796, 0.850] p=0.76	0.734 [0.719, 0.749] p=0.17
One DF branch method (IRCSN)	0.745 [0.732, 0.759] p≪0.01	0.839 [0.817, 0.861] p=0.92	0.713 [0.702, 0.724] p<0.01
Two DF branches method (I3D+ IRCSN)	0.748 [0.734, 0.761] p≪0.01	0.813 [0.792, 0.835] p=0.34	0.717 [0.704, 0.730] p=0.01
Two DF branches method (IRCSN+SlowFast)	0.769 [0.757, 0.780] p=0.01	0.821 [0.796, 0.847] p=0.57	0.724 [0.712, 0.735] p=0.04
Three DF branches method (I3D+ IRCSN+SlowFast)	0.774 [0.761, 0.786] p=0.05	0.802 [0.771, 0.832] p=0.20	0.722 [0.707, 0.738] p=0.05

*Note*: The proposed method achieved the best performance when radiomic features and deep features from the Inflated 3D ConvNet and SlowFast Networks were included in the network.

^a^ 95% confidence intervals (CI) of variable.

* *p* value of for Wilcoxon rank‐sum test and p≪0.01 means $p<10^{-4}$.

### 3.2 Beneficial of combining features into classifier

This study seeks to elucidate the marginal effect of integrating features from diverse modalities into a classification model. Table 1 demonstrates that both deep learning features and radiomics significantly enhance the model's performance. Comparative analyses indicate that models relying exclusively on radiomics^39^ or deep features^13^ are worse than those utilizing a combination of both. Specifically, models integrating hybrid features [deep features (DF) based method (I3D)] markedly outperform the pure radiomics and deep learning features based, as confirmed by statistically significant results (*p* < 0.01 and *p*≪0.01, respectively) in the Wilcoxon rank‐sum test. Nonetheless, incorporating deep features from IRCSN networks does not appear to positively impact classifier performance.

Another aspect this study investigates is the marginal effect of amalgamating multiple deep features from disparate networks. The experimental findings suggest that combining deep features from the I3D and Slow Fast networks yields the best outcomes, signifying that the integration of these specific deep features is consequential. However, employing deep features from IRCSN networks as an additional branch does not enhance model performance. On the contrary, integrating features from IRCSN leads to a decrease in classifier efficacy (One DF Branch Method—I3D vs. Two DF Branches Method—I3D+ IRCSN, p<0.01; One DF Branch Method—SlowFast vs. Two DF Branches Method—SlowFast + IRCSN, p=0.68; Proposed Method—I3D+ SlowFast vs. Three DF Branches Method, p=0.05), despite IRCSN's superior performance in action recognition among the three networks. The potential explanations for this discrepancy will be discussed in the Discussion section.

### 3.3 External validation results in the H&N 1 dataset

External validation results of proposed method and reference methods in H&N1 dataset^13^, ^39^ finished, results were shown in Table 2, a notable decrease in performance was observed in several models. Specifically, the deep feature based method^39^ achieved an AUC of 0.700 (95% CI, [0.682–0.717]), along with a recall of 0.907 (95% CI, [0.870–0.945]) and accuracy of 0.473 (95% CI, [0.442–0.503]) during external validation. A potential reason for the diminished recall could be the low calibration accuracy of the established method, which appears to be overconfident in its results —a topic explored in further studies.^40^ In summary, methods based on deep features demonstrated robust performance in external validation.

**TABLE 2:** External validation results of proposed method and reference methods in H&N1.

Methods/Metrics	AUC	Recall	Accuracy
Proposed method (I3D+ SlowFast)	0.581 [0.560,0.603] p≪0.01 ^*^	0.664 [0.832, 0.863] p≪0.01	0.520 [0.467,0.573] p≪0.01
Hand‐crafted features‐based method^29^	0.549 [0.544,0.554] p≪0.01	0.696 [0.549, 0.842] p≪0.01	0.483 [0.422,0.543] p≪0.01
Deep features (DF) based method (I3D)^13^	0.700 [0.682, 0.717] p=0.29	0.907 [0.870, 0.945] p=0.03	0.473 [0.442,0.503] p≪0.01

*Note*: A notable decrease in performance was observed for the classifier in the external validation, with a more significant decline seen in the radiomics‐based model compared to the deep feature‐based network.

* *p* value of for Wilcoxon rank‐sum test compared with internal validation results.

Moreover, AUC of proposed method and radiomics based Method^13^ decreased to 0.581 (95% CI, [0.560–0.603]) and 0.549 (95% CI, [0.544–0.554]), respectively, with similar significant declines observed in recall and accuracy. The decrease in performance was more pronounced for the radiomics‐based method compared to the proposed method.

A preliminary conclusion suggests that classifiers based on deep features may be more resilient than those reliant on hand‐crafted features during external validation, with radiomics features potentially exerting a detrimental effect on classifier performance. The significant performance decline of methods based on radiomics features during external validation, along with potential strategies to mitigate this issue, will be discussed in the Discussion section. Despite the observed underperformance of radiomics‐based methods in external validation, it is argued that they should not be excluded from classifiers, with supporting arguments to be detailed in the Discussion section.

### 3.4 Ablation studies

**FIGURE 5:**
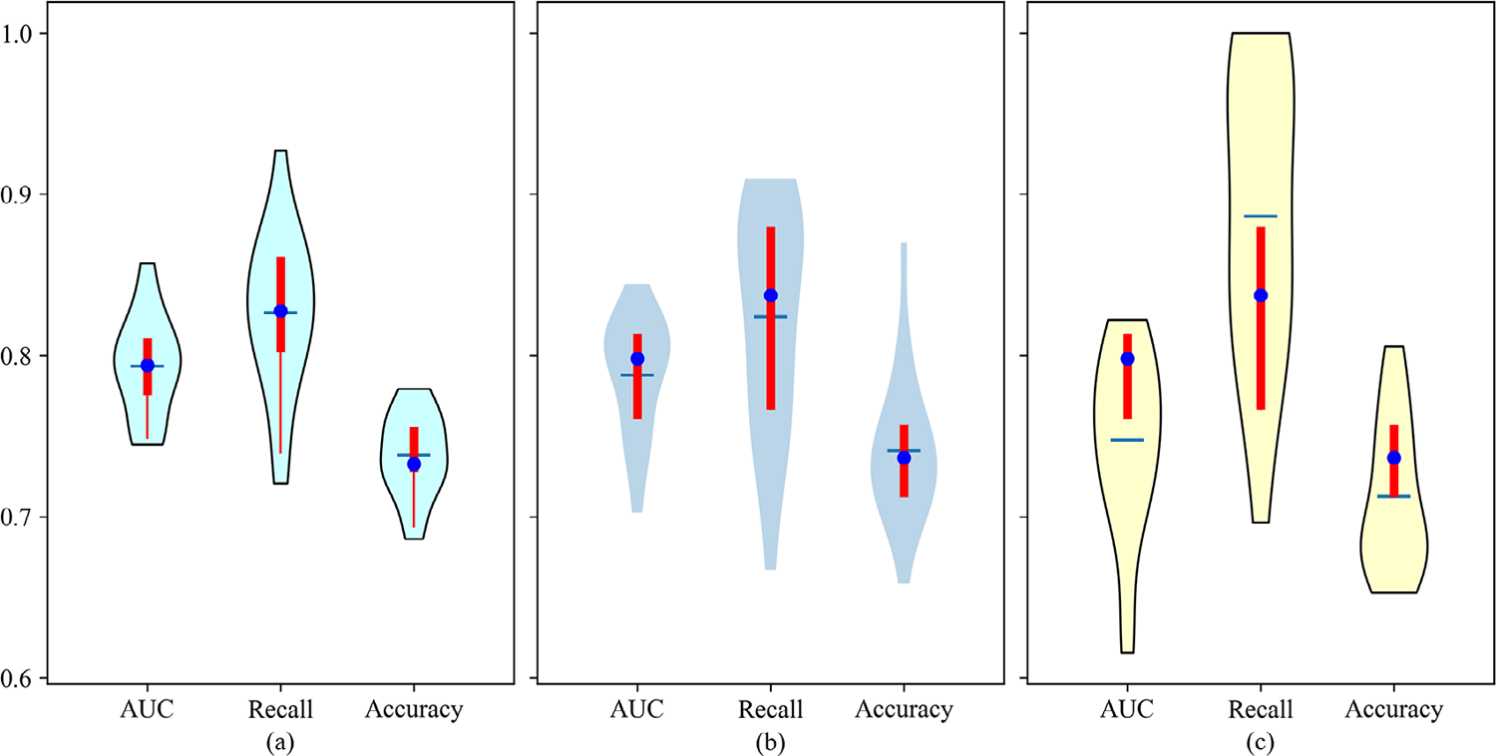
Violins plot of ablation study of scanners for HPV prediction classifier. (a) Results of classifier based on whole RADCURE dataset without manufacturer normalization; (b) Results of classifier based on image samples collected from TOSHIBA scanners in RADCURE dataset (1029 valid samples); (c) Results of classifier based on image samples collected from GE MEDICAL SYSTEMS (363 valid samples).

The ablation study examining the impact of scanner bias on classifier performance is presented in Figure 5. The proposed method achieved an AUC of 0.785 [95% CI, (0.766–0.805)] using image samples from TOSHIBA scanners, and an AUC of 0.745 [95% CI, (0.721–0.774)] using images from GE MEDICAL SYSTEMS. In contrast, when evaluated on the entire dataset without manufacturer normalization, the proposed method yielded an AUC of 0.774 [95% CI, (0.760–0.789)]. Results show that scanner bias effect for the classifier is significant (*p* < 0.05) for all three metrics and introducing manufacturer normalization is effective for classifier. Regarding the competitive results of methods in TOSHIBA‐only images, features derived from homogeneous data demonstrated more consistent performance and higher efficacy in certain tasks.

As previously discussed, manufacturer normalization was implemented to counteract the scanner bias inherent in the data. An ablation study examining the efficacy of this normalization technique on both the proposed and reference methods was conducted, with findings presented in Table 3. The results demonstrate that manufacturer normalization significantly enhances classifier performance, particularly for methods based on radiomics feature, a finding corroborated by other studies.^41^ However, it appears that manufacturer normalization does not markedly improve the performance of classifiers utilizing deep features. This may be attributed to the nature of deep features as high‐level features, which are presumably less affected by scanner bias compared to lower‐level features, such as radiomics.

**TABLE 3:** Ablation study results of manufacturer normalization for proposed method and reference methods.

Methods/Metrics	AUC	Recall	Accuracy
Proposed Method (I3D+ SlowFast)	0.774 [0.760,0.789] p=0.06 ^*^	0.807 [0.784, 0.831] p=0.18	0.719 [0.707,0.732] p=0.06
Hand‐crafted features‐based method^39^	0.724 [0.713,0.735] p<0.01	0.826 [0.807, 0.844] p=0.04	0.689 [0.677,0.702] p=0.03
Deep features (DF) based method (I3D)^13^	0.698 [0.688, 0.710] p=0.04	0.837 [0.805, 0.868] p=0.50	0.685 [0.658,0.713] p=0.18

* *p* value of for Wilcoxon rank‐sum test compared with method with manufacturer normalization.

## 4 DISCUSSION

HPV infection has been recognized as a significant risk factor for oropharyngeal cancers. In clinical practice, the methods commonly used for HPV detection are often time‐consuming and sometimes invasive. This study introduces a novel predictive approach for identifying HPV presence in oropharyngeal cancers through CT imaging.

The rationale for extracting deep features from the output layer of a pre‐trained natural video action recognition network is detailed in previous published study.^20^, ^42^ The summary of this rationale includes the absence of a multi‐class classification network trained on 3D medical images, prompting the use of a video‐trained network as an interim solution. Features from the output layer were preferred over those from fully connected layers due to their enhanced interpretability and reproducibility. The decision to utilize features from a video‐trained network rather than those from domain‐specific encoder‐decoder networks (e.g., CT, MRI) was based on the lack of conclusive evidence favoring the performance of encoder‐decoder network‐derived features over those trained on natural images (e.g., ImageNet and video data) within an existing multi‐class classification model.^43^

The performance of used pretrained networks for action recognition tasks can be found on some open‐access websites,^44^ although the IRCSN network exhibited superior performance in action recognition, it demonstrated suboptimal results when its deep features were used as a supplementary branch. Possible explanations for this include IRCSN's overfitting to the “Kinetics 400 dataset” and a significant texture gap between the “Kinetics 400 dataset” and the data used in this study, potentially diminishing the IRCSN's ability to represent information effectively. Additionally, the difference in activation functions between the output layers of the networks used may contribute to this phenomenon. Specifically, the IRCSN network employs a linear activation function, which yields smaller differentiation in output values, a limitation that becomes more pronounced when applied to data from a different domain, unlike the Sigmoid activation function used in the I3D and Slow Fast networks.

SNNs form the core of our classifier. They consist of two identical subnetworks that share weights and process input pairs to learn their similarity. The networks generate embeddings, which are then compared using a distance metric to determine whether the inputs are similar.^45^ The unique characteristics and advantages of SNNs have accelerated their application in various tasks, including face verification,^46^ signature verification,^47^ one‐shot learning,^48^ and image retrieval,^49^ among others.

This study investigates potential reasons for the suboptimal performance of the proposed method during external validation, with a focus on the vulnerability of radiomics features across different datasets. Radiomics features, being low‐level features extracted directly from imaging data, are notably sensitive to dataset imaging biases, a challenge extensively discussed in the literature.^50^ In contrast, deep features are high‐level features derived from the advanced layers of a network, demonstrating greater robustness to imaging biases. Further research into the performance disparities between classifiers based on deep features and those based on handcrafted features during external validation presents an intriguing avenue for future investigation.

Besides the vulnerability of features, overfitting is a significant factor contributing to performance degradation during internal and external validation, particularly for radiomics‐based classifiers^20^, ^51^ Addressing this limitation and reducing the incidence of overfitting is a crucial and compelling area for future research, and several solutions have been proposed in recent years. One potential approach to enhance classifier performance across datasets is feature engineering‐based feature selection prior to classifier construction, which has demonstrated effectiveness in several studies^52^, ^53^ Another promising solution is the normalization of multicenter datasets using generative models. Techniques such as generative adversarial networks and diffusion models have shown potential, with several pioneering studies published.^54^, ^55^

The decision not to exclude radiomics features from the classifier, despite their significant negative impact during external validation, is twofold. First, ablation studies have highlighted the effectiveness of radiomics, with some models outperforming those based solely on a single deep feature branch. Second, the interpretability and human comprehensibility of models based on radiomics features currently surpass those of deep feature based methods, an essential consideration for computer‐aided diagnosis systems. Thus, efforts should focus on enhancing the external validation performance of radiomics feature‐based classifiers rather than their abandonment in the era of deep learning.^56^

Regarding the limitations of this study, class imbalance in the HPV status of both our internal and external validation datasets may reduce the performance of our model. Specifically, the performance degradation in classification models arises from their tendency to favor the majority class due to its larger representation in the dataset, leading to biased predictions.^57^ This bias can hinder the model's ability to generalize to the minority class, where it struggles to learn the relevant features, potentially resulting in misleading performance metrics, such as accuracy, which may not accurately reflect true model performance.^58^ Consequently, the minority class is often underrepresented in the decision boundary, increasing the risk of misclassifying critical instances, particularly in applications like medical diagnosis or fraud detection. To mitigate this issue, techniques such as resampling, class weighting, and alternative evaluation metrics (e.g., precision, recall, and F1 score) are commonly employed.^59^

Additionally, the potential truncation errors arising from converting DICOM data to the input format for action recognition networks, along with possibly inappropriate window width and level settings due to imaging collection protocol biases, must be acknowledged. Then, the 3D deep features were not extracted using SOTA networks for action recognition, reflecting the exploratory nature of this study rather than a quest for the optimal structure. Notably, the suitability of deep features from networks excelling in action recognition for this specific task is not guaranteed, as evidenced by the poor performance of features from the IRCSN network. The interpretability of the proposed method is another challenge, with conventional explainable artificial intelligence (XAI) approaches proving ineffective, especially when incorporating high‐level features like deep features. Continuous efforts to improve classifier performance in external validation, particularly for studies involving radiomics, are imperative. Finally, the validation and external validation results demonstrate the effectiveness of the proposed framework. However, the validity of our conclusions may be compromised if changes were made to the selected model, given the potential for dataset bias and classifier preferences. Further investigation into this issue would be valuable.

## 5 CONCLUSION

This study introduces a novel predictive methodology for detecting HPV presence in oropharyngeal cancers through CT imaging. Specifically, we propose a model utilizing a SNN architecture. This model integrates multi‐modality off‐the‐shelf features—handcrafted features and 3D deep features—as inputs to enhance information representation. To mitigate scanner bias, manufacturer normalization was applied, and external validation was conducted to bolster the results’ reliability. Our design philosophy emphasized transfer learning to alleviate computational demands and enhance the method's accessibility for clinical practitioners. The outcomes demonstrate that our proposed method surpasses existing models in performance and can be efficiently implemented on a single CPU‐based platform, significantly reducing computational resource requirements for clinical users.

## AUTHOR CONTRIBUTIONS

**Junhua Chen**: Conceptualization; methodology; formal analysis; investigation; writing—original draft; writing—review & editing. **Yanyan Cheng**: Methodology; investigation; writing—review & editing. **Lijun Chen**: Methodology; formal analysis; writing—review & editing. **Banghua Yang**: Conceptualization; writing—review & editing; supervision.

## CONFLICT OF INTEREST STATEMENT

The authors declare no conflicts of interest.

## Supporting information



Supporting information
