# Supplementary material for: Human papillomavirus (HPV) prediction for oropharyngeal cancer based on CT by using off‐the‐shelf features: A dual‐dataset study
Source: J Appl Clin Med Phys. 2025 Mar 2;26(5):e70061. doi: 10.1002/acm2.70061 (PMC12059277; doi:10.1002/acm2.70061)
Supplement: Supplementary file 1 — Supporting information [file ACM2-26-e70061-s001.docx]

Table S1. index of patients eligible for inclusion in the RADCURE dataset

| RADCURE-0005 | RADCURE-0517 | RADCURE-1025 | RADCURE-1855 | RADCURE-2472 | RADCURE-2868 | RADCURE-3296 | RADCURE-3631 |
| --- | --- | --- | --- | --- | --- | --- | --- |
| RADCURE-0007 | RADCURE-0521 | RADCURE-1026 | RADCURE-1859 | RADCURE-2473 | RADCURE-2870 | RADCURE-3297 | RADCURE-3632 |
| RADCURE-0010 | RADCURE-0522 | RADCURE-1033 | RADCURE-1862 | RADCURE-2474 | RADCURE-2872 | RADCURE-3298 | RADCURE-3634 |
| RADCURE-0017 | RADCURE-0524 | RADCURE-1035 | RADCURE-1864 | RADCURE-2479 | RADCURE-2874 | RADCURE-3299 | RADCURE-3637 |
| RADCURE-0021 | RADCURE-0529 | RADCURE-1038 | RADCURE-1871 | RADCURE-2484 | RADCURE-2879 | RADCURE-3301 | RADCURE-3638 |
| RADCURE-0022 | RADCURE-0531 | RADCURE-1040 | RADCURE-1872 | RADCURE-2485 | RADCURE-2882 | RADCURE-3302 | RADCURE-3639 |
| RADCURE-0025 | RADCURE-0535 | RADCURE-1045 | RADCURE-1879 | RADCURE-2487 | RADCURE-2884 | RADCURE-3303 | RADCURE-3640 |
| RADCURE-0030 | RADCURE-0536 | RADCURE-1047 | RADCURE-1882 | RADCURE-2489 | RADCURE-2886 | RADCURE-3305 | RADCURE-3641 |
| RADCURE-0031 | RADCURE-0544 | RADCURE-1048 | RADCURE-1885 | RADCURE-2490 | RADCURE-2889 | RADCURE-3307 | RADCURE-3644 |
| RADCURE-0035 | RADCURE-0546 | RADCURE-1051 | RADCURE-1890 | RADCURE-2491 | RADCURE-2890 | RADCURE-3308 | RADCURE-3645 |
| RADCURE-0036 | RADCURE-0548 | RADCURE-1052 | RADCURE-1891 | RADCURE-2493 | RADCURE-2891 | RADCURE-3310 | RADCURE-3646 |
| RADCURE-0037 | RADCURE-0550 | RADCURE-1056 | RADCURE-1896 | RADCURE-2497 | RADCURE-2893 | RADCURE-3311 | RADCURE-3648 |
| RADCURE-0043 | RADCURE-0552 | RADCURE-1057 | RADCURE-1898 | RADCURE-2498 | RADCURE-2897 | RADCURE-3313 | RADCURE-3649 |
| RADCURE-0049 | RADCURE-0553 | RADCURE-1059 | RADCURE-1902 | RADCURE-2500 | RADCURE-2898 | RADCURE-3317 | RADCURE-3651 |
| RADCURE-0053 | RADCURE-0557 | RADCURE-1060 | RADCURE-1916 | RADCURE-2505 | RADCURE-2903 | RADCURE-3318 | RADCURE-3652 |
| RADCURE-0057 | RADCURE-0559 | RADCURE-1061 | RADCURE-1925 | RADCURE-2507 | RADCURE-2904 | RADCURE-3319 | RADCURE-3656 |
| RADCURE-0059 | RADCURE-0560 | RADCURE-1069 | RADCURE-1927 | RADCURE-2508 | RADCURE-2905 | RADCURE-3320 | RADCURE-3657 |
| RADCURE-0060 | RADCURE-0562 | RADCURE-1070 | RADCURE-1931 | RADCURE-2509 | RADCURE-2907 | RADCURE-3322 | RADCURE-3658 |
| RADCURE-0062 | RADCURE-0563 | RADCURE-1073 | RADCURE-1932 | RADCURE-2511 | RADCURE-2908 | RADCURE-3325 | RADCURE-3660 |
| RADCURE-0065 | RADCURE-0564 | RADCURE-1075 | RADCURE-1939 | RADCURE-2516 | RADCURE-2909 | RADCURE-3328 | RADCURE-3661 |
| RADCURE-0070 | RADCURE-0568 | RADCURE-1089 | RADCURE-1949 | RADCURE-2523 | RADCURE-2912 | RADCURE-3329 | RADCURE-3663 |
| RADCURE-0071 | RADCURE-0573 | RADCURE-1090 | RADCURE-1950 | RADCURE-2524 | RADCURE-2913 | RADCURE-3330 | RADCURE-3665 |
| RADCURE-0076 | RADCURE-0574 | RADCURE-1096 | RADCURE-1951 | RADCURE-2525 | RADCURE-2916 | RADCURE-3331 | RADCURE-3667 |
| RADCURE-0077 | RADCURE-0583 | RADCURE-1118 | RADCURE-1958 | RADCURE-2528 | RADCURE-2917 | RADCURE-3333 | RADCURE-3668 |
| RADCURE-0080 | RADCURE-0586 | RADCURE-1156 | RADCURE-1961 | RADCURE-2529 | RADCURE-2919 | RADCURE-3334 | RADCURE-3671 |
| RADCURE-0086 | RADCURE-0590 | RADCURE-1177 | RADCURE-1963 | RADCURE-2530 | RADCURE-2926 | RADCURE-3337 | RADCURE-3674 |
| RADCURE-0089 | RADCURE-0594 | RADCURE-1179 | RADCURE-1965 | RADCURE-2532 | RADCURE-2927 | RADCURE-3340 | RADCURE-3679 |
| RADCURE-0090 | RADCURE-0596 | RADCURE-1187 | RADCURE-1970 | RADCURE-2535 | RADCURE-2930 | RADCURE-3341 | RADCURE-3683 |
| RADCURE-0091 | RADCURE-0600 | RADCURE-1197 | RADCURE-1986 | RADCURE-2538 | RADCURE-2931 | RADCURE-3348 | RADCURE-3684 |
| RADCURE-0092 | RADCURE-0605 | RADCURE-1205 | RADCURE-1991 | RADCURE-2539 | RADCURE-2932 | RADCURE-3349 | RADCURE-3686 |
| RADCURE-0098 | RADCURE-0607 | RADCURE-1220 | RADCURE-1993 | RADCURE-2540 | RADCURE-2933 | RADCURE-3351 | RADCURE-3687 |
| RADCURE-0104 | RADCURE-0608 | RADCURE-1235 | RADCURE-1999 | RADCURE-2545 | RADCURE-2934 | RADCURE-3353 | RADCURE-3689 |
| RADCURE-0105 | RADCURE-0611 | RADCURE-1242 | RADCURE-2000 | RADCURE-2546 | RADCURE-2937 | RADCURE-3354 | RADCURE-3691 |
| RADCURE-0106 | RADCURE-0612 | RADCURE-1254 | RADCURE-2002 | RADCURE-2551 | RADCURE-2939 | RADCURE-3357 | RADCURE-3692 |
| RADCURE-0107 | RADCURE-0616 | RADCURE-1259 | RADCURE-2008 | RADCURE-2555 | RADCURE-2940 | RADCURE-3359 | RADCURE-3695 |
| RADCURE-0110 | RADCURE-0621 | RADCURE-1261 | RADCURE-2010 | RADCURE-2556 | RADCURE-2943 | RADCURE-3360 | RADCURE-3696 |
| RADCURE-0112 | RADCURE-0622 | RADCURE-1262 | RADCURE-2012 | RADCURE-2559 | RADCURE-2945 | RADCURE-3361 | RADCURE-3698 |
| RADCURE-0117 | RADCURE-0627 | RADCURE-1277 | RADCURE-2015 | RADCURE-2567 | RADCURE-2948 | RADCURE-3362 | RADCURE-3702 |
| RADCURE-0124 | RADCURE-0628 | RADCURE-1278 | RADCURE-2017 | RADCURE-2569 | RADCURE-2950 | RADCURE-3364 | RADCURE-3703 |
| RADCURE-0128 | RADCURE-0629 | RADCURE-1284 | RADCURE-2019 | RADCURE-2570 | RADCURE-2951 | RADCURE-3366 | RADCURE-3707 |
| RADCURE-0129 | RADCURE-0631 | RADCURE-1286 | RADCURE-2020 | RADCURE-2571 | RADCURE-2953 | RADCURE-3368 | RADCURE-3711 |
| RADCURE-0134 | RADCURE-0632 | RADCURE-1289 | RADCURE-2039 | RADCURE-2572 | RADCURE-2954 | RADCURE-3371 | RADCURE-3712 |
| RADCURE-0136 | RADCURE-0633 | RADCURE-1290 | RADCURE-2040 | RADCURE-2576 | RADCURE-2956 | RADCURE-3373 | RADCURE-3713 |
| RADCURE-0140 | RADCURE-0634 | RADCURE-1292 | RADCURE-2041 | RADCURE-2577 | RADCURE-2958 | RADCURE-3374 | RADCURE-3717 |
| RADCURE-0141 | RADCURE-0636 | RADCURE-1297 | RADCURE-2046 | RADCURE-2578 | RADCURE-2964 | RADCURE-3378 | RADCURE-3719 |
| RADCURE-0144 | RADCURE-0638 | RADCURE-1302 | RADCURE-2063 | RADCURE-2579 | RADCURE-2965 | RADCURE-3379 | RADCURE-3721 |
| RADCURE-0146 | RADCURE-0639 | RADCURE-1307 | RADCURE-2066 | RADCURE-2580 | RADCURE-2966 | RADCURE-3382 | RADCURE-3726 |
| RADCURE-0147 | RADCURE-0640 | RADCURE-1313 | RADCURE-2067 | RADCURE-2581 | RADCURE-2968 | RADCURE-3385 | RADCURE-3727 |
| RADCURE-0148 | RADCURE-0644 | RADCURE-1315 | RADCURE-2073 | RADCURE-2583 | RADCURE-2971 | RADCURE-3386 | RADCURE-3729 |
| RADCURE-0157 | RADCURE-0648 | RADCURE-1321 | RADCURE-2075 | RADCURE-2584 | RADCURE-2975 | RADCURE-3387 | RADCURE-3732 |
| RADCURE-0158 | RADCURE-0652 | RADCURE-1322 | RADCURE-2076 | RADCURE-2585 | RADCURE-2979 | RADCURE-3389 | RADCURE-3736 |
| RADCURE-0159 | RADCURE-0653 | RADCURE-1333 | RADCURE-2079 | RADCURE-2586 | RADCURE-2981 | RADCURE-3390 | RADCURE-3740 |
| RADCURE-0167 | RADCURE-0655 | RADCURE-1334 | RADCURE-2081 | RADCURE-2590 | RADCURE-2982 | RADCURE-3392 | RADCURE-3741 |
| RADCURE-0170 | RADCURE-0657 | RADCURE-1335 | RADCURE-2085 | RADCURE-2591 | RADCURE-2985 | RADCURE-3395 | RADCURE-3744 |
| RADCURE-0171 | RADCURE-0658 | RADCURE-1341 | RADCURE-2088 | RADCURE-2592 | RADCURE-2987 | RADCURE-3396 | RADCURE-3748 |
| RADCURE-0176 | RADCURE-0661 | RADCURE-1353 | RADCURE-2091 | RADCURE-2595 | RADCURE-2988 | RADCURE-3397 | RADCURE-3751 |
| RADCURE-0183 | RADCURE-0662 | RADCURE-1355 | RADCURE-2093 | RADCURE-2596 | RADCURE-2990 | RADCURE-3399 | RADCURE-3752 |
| RADCURE-0186 | RADCURE-0665 | RADCURE-1360 | RADCURE-2106 | RADCURE-2597 | RADCURE-2992 | RADCURE-3400 | RADCURE-3754 |
| RADCURE-0187 | RADCURE-0668 | RADCURE-1362 | RADCURE-2107 | RADCURE-2601 | RADCURE-2993 | RADCURE-3401 | RADCURE-3755 |
| RADCURE-0188 | RADCURE-0670 | RADCURE-1365 | RADCURE-2108 | RADCURE-2603 | RADCURE-2996 | RADCURE-3402 | RADCURE-3756 |
| RADCURE-0191 | RADCURE-0674 | RADCURE-1376 | RADCURE-2110 | RADCURE-2604 | RADCURE-3000 | RADCURE-3403 | RADCURE-3758 |
| RADCURE-0195 | RADCURE-0677 | RADCURE-1378 | RADCURE-2114 | RADCURE-2607 | RADCURE-3002 | RADCURE-3404 | RADCURE-3759 |
| RADCURE-0196 | RADCURE-0679 | RADCURE-1381 | RADCURE-2118 | RADCURE-2613 | RADCURE-3005 | RADCURE-3405 | RADCURE-3760 |
| RADCURE-0198 | RADCURE-0683 | RADCURE-1382 | RADCURE-2120 | RADCURE-2616 | RADCURE-3006 | RADCURE-3414 | RADCURE-3765 |
| RADCURE-0199 | RADCURE-0687 | RADCURE-1383 | RADCURE-2128 | RADCURE-2617 | RADCURE-3007 | RADCURE-3420 | RADCURE-3766 |
| RADCURE-0201 | RADCURE-0689 | RADCURE-1387 | RADCURE-2134 | RADCURE-2618 | RADCURE-3008 | RADCURE-3421 | RADCURE-3770 |
| RADCURE-0203 | RADCURE-0690 | RADCURE-1388 | RADCURE-2142 | RADCURE-2621 | RADCURE-3011 | RADCURE-3425 | RADCURE-3772 |
| RADCURE-0204 | RADCURE-0699 | RADCURE-1390 | RADCURE-2144 | RADCURE-2624 | RADCURE-3013 | RADCURE-3426 | RADCURE-3774 |
| RADCURE-0206 | RADCURE-0702 | RADCURE-1409 | RADCURE-2152 | RADCURE-2625 | RADCURE-3014 | RADCURE-3427 | RADCURE-3775 |
| RADCURE-0207 | RADCURE-0703 | RADCURE-1410 | RADCURE-2154 | RADCURE-2626 | RADCURE-3018 | RADCURE-3428 | RADCURE-3777 |
| RADCURE-0210 | RADCURE-0704 | RADCURE-1436 | RADCURE-2155 | RADCURE-2627 | RADCURE-3020 | RADCURE-3429 | RADCURE-3778 |
| RADCURE-0214 | RADCURE-0709 | RADCURE-1462 | RADCURE-2158 | RADCURE-2628 | RADCURE-3025 | RADCURE-3430 | RADCURE-3782 |
| RADCURE-0215 | RADCURE-0711 | RADCURE-1465 | RADCURE-2167 | RADCURE-2630 | RADCURE-3034 | RADCURE-3431 | RADCURE-3783 |
| RADCURE-0222 | RADCURE-0712 | RADCURE-1467 | RADCURE-2168 | RADCURE-2631 | RADCURE-3035 | RADCURE-3435 | RADCURE-3784 |
| RADCURE-0226 | RADCURE-0718 | RADCURE-1470 | RADCURE-2170 | RADCURE-2632 | RADCURE-3036 | RADCURE-3437 | RADCURE-3787 |
| RADCURE-0227 | RADCURE-0723 | RADCURE-1475 | RADCURE-2171 | RADCURE-2635 | RADCURE-3038 | RADCURE-3441 | RADCURE-3790 |
| RADCURE-0228 | RADCURE-0729 | RADCURE-1479 | RADCURE-2173 | RADCURE-2636 | RADCURE-3042 | RADCURE-3442 | RADCURE-3791 |
| RADCURE-0231 | RADCURE-0736 | RADCURE-1482 | RADCURE-2175 | RADCURE-2637 | RADCURE-3043 | RADCURE-3445 | RADCURE-3792 |
| RADCURE-0232 | RADCURE-0737 | RADCURE-1483 | RADCURE-2177 | RADCURE-2638 | RADCURE-3044 | RADCURE-3446 | RADCURE-3793 |
| RADCURE-0233 | RADCURE-0742 | RADCURE-1488 | RADCURE-2181 | RADCURE-2640 | RADCURE-3046 | RADCURE-3448 | RADCURE-3799 |
| RADCURE-0235 | RADCURE-0743 | RADCURE-1489 | RADCURE-2187 | RADCURE-2642 | RADCURE-3052 | RADCURE-3449 | RADCURE-3801 |
| RADCURE-0239 | RADCURE-0744 | RADCURE-1497 | RADCURE-2190 | RADCURE-2644 | RADCURE-3054 | RADCURE-3450 | RADCURE-3802 |
| RADCURE-0240 | RADCURE-0745 | RADCURE-1500 | RADCURE-2193 | RADCURE-2649 | RADCURE-3061 | RADCURE-3451 | RADCURE-3803 |
| RADCURE-0242 | RADCURE-0750 | RADCURE-1503 | RADCURE-2194 | RADCURE-2656 | RADCURE-3063 | RADCURE-3452 | RADCURE-3804 |
| RADCURE-0244 | RADCURE-0751 | RADCURE-1504 | RADCURE-2195 | RADCURE-2657 | RADCURE-3064 | RADCURE-3453 | RADCURE-3806 |
| RADCURE-0246 | RADCURE-0752 | RADCURE-1505 | RADCURE-2203 | RADCURE-2658 | RADCURE-3065 | RADCURE-3457 | RADCURE-3807 |
| RADCURE-0247 | RADCURE-0760 | RADCURE-1509 | RADCURE-2208 | RADCURE-2659 | RADCURE-3066 | RADCURE-3459 | RADCURE-3809 |
| RADCURE-0249 | RADCURE-0761 | RADCURE-1511 | RADCURE-2209 | RADCURE-2660 | RADCURE-3068 | RADCURE-3461 | RADCURE-3810 |
| RADCURE-0250 | RADCURE-0763 | RADCURE-1512 | RADCURE-2210 | RADCURE-2664 | RADCURE-3072 | RADCURE-3462 | RADCURE-3814 |
| RADCURE-0257 | RADCURE-0766 | RADCURE-1516 | RADCURE-2211 | RADCURE-2665 | RADCURE-3075 | RADCURE-3463 | RADCURE-3816 |
| RADCURE-0259 | RADCURE-0767 | RADCURE-1523 | RADCURE-2212 | RADCURE-2666 | RADCURE-3081 | RADCURE-3464 | RADCURE-3818 |
| RADCURE-0260 | RADCURE-0769 | RADCURE-1528 | RADCURE-2213 | RADCURE-2668 | RADCURE-3084 | RADCURE-3465 | RADCURE-3821 |
| RADCURE-0261 | RADCURE-0775 | RADCURE-1529 | RADCURE-2215 | RADCURE-2669 | RADCURE-3085 | RADCURE-3467 | RADCURE-3822 |
| RADCURE-0263 | RADCURE-0778 | RADCURE-1539 | RADCURE-2220 | RADCURE-2671 | RADCURE-3086 | RADCURE-3468 | RADCURE-3824 |
| RADCURE-0267 | RADCURE-0782 | RADCURE-1543 | RADCURE-2223 | RADCURE-2679 | RADCURE-3090 | RADCURE-3471 | RADCURE-3825 |
| RADCURE-0270 | RADCURE-0783 | RADCURE-1545 | RADCURE-2229 | RADCURE-2681 | RADCURE-3094 | RADCURE-3472 | RADCURE-3828 |
| RADCURE-0277 | RADCURE-0786 | RADCURE-1547 | RADCURE-2230 | RADCURE-2682 | RADCURE-3095 | RADCURE-3473 | RADCURE-3831 |
| RADCURE-0279 | RADCURE-0787 | RADCURE-1549 | RADCURE-2232 | RADCURE-2683 | RADCURE-3096 | RADCURE-3477 | RADCURE-3837 |
| RADCURE-0280 | RADCURE-0791 | RADCURE-1551 | RADCURE-2234 | RADCURE-2684 | RADCURE-3098 | RADCURE-3478 | RADCURE-3838 |
| RADCURE-0282 | RADCURE-0792 | RADCURE-1554 | RADCURE-2241 | RADCURE-2686 | RADCURE-3103 | RADCURE-3481 | RADCURE-3841 |
| RADCURE-0284 | RADCURE-0794 | RADCURE-1555 | RADCURE-2244 | RADCURE-2688 | RADCURE-3104 | RADCURE-3482 | RADCURE-3842 |
| RADCURE-0285 | RADCURE-0795 | RADCURE-1557 | RADCURE-2248 | RADCURE-2689 | RADCURE-3105 | RADCURE-3483 | RADCURE-3843 |
| RADCURE-0286 | RADCURE-0796 | RADCURE-1560 | RADCURE-2250 | RADCURE-2690 | RADCURE-3109 | RADCURE-3485 | RADCURE-3844 |
| RADCURE-0289 | RADCURE-0800 | RADCURE-1562 | RADCURE-2251 | RADCURE-2692 | RADCURE-3111 | RADCURE-3486 | RADCURE-3846 |
| RADCURE-0290 | RADCURE-0802 | RADCURE-1565 | RADCURE-2252 | RADCURE-2696 | RADCURE-3113 | RADCURE-3487 | RADCURE-3850 |
| RADCURE-0293 | RADCURE-0803 | RADCURE-1574 | RADCURE-2253 | RADCURE-2702 | RADCURE-3114 | RADCURE-3488 | RADCURE-3852 |
| RADCURE-0298 | RADCURE-0806 | RADCURE-1575 | RADCURE-2256 | RADCURE-2704 | RADCURE-3115 | RADCURE-3492 | RADCURE-3855 |
| RADCURE-0299 | RADCURE-0809 | RADCURE-1583 | RADCURE-2257 | RADCURE-2707 | RADCURE-3119 | RADCURE-3493 | RADCURE-3858 |
| RADCURE-0300 | RADCURE-0810 | RADCURE-1584 | RADCURE-2260 | RADCURE-2708 | RADCURE-3120 | RADCURE-3494 | RADCURE-3861 |
| RADCURE-0307 | RADCURE-0813 | RADCURE-1585 | RADCURE-2261 | RADCURE-2710 | RADCURE-3132 | RADCURE-3496 | RADCURE-3863 |
| RADCURE-0313 | RADCURE-0817 | RADCURE-1586 | RADCURE-2262 | RADCURE-2712 | RADCURE-3136 | RADCURE-3497 | RADCURE-3864 |
| RADCURE-0315 | RADCURE-0819 | RADCURE-1591 | RADCURE-2263 | RADCURE-2714 | RADCURE-3137 | RADCURE-3498 | RADCURE-3866 |
| RADCURE-0320 | RADCURE-0825 | RADCURE-1603 | RADCURE-2265 | RADCURE-2720 | RADCURE-3139 | RADCURE-3499 | RADCURE-3867 |
| RADCURE-0322 | RADCURE-0829 | RADCURE-1604 | RADCURE-2266 | RADCURE-2722 | RADCURE-3140 | RADCURE-3500 | RADCURE-3868 |
| RADCURE-0323 | RADCURE-0832 | RADCURE-1605 | RADCURE-2270 | RADCURE-2727 | RADCURE-3145 | RADCURE-3501 | RADCURE-3869 |
| RADCURE-0326 | RADCURE-0834 | RADCURE-1606 | RADCURE-2282 | RADCURE-2729 | RADCURE-3146 | RADCURE-3504 | RADCURE-3875 |
| RADCURE-0327 | RADCURE-0838 | RADCURE-1608 | RADCURE-2286 | RADCURE-2730 | RADCURE-3149 | RADCURE-3505 | RADCURE-3876 |
| RADCURE-0329 | RADCURE-0841 | RADCURE-1614 | RADCURE-2287 | RADCURE-2731 | RADCURE-3151 | RADCURE-3506 | RADCURE-3880 |
| RADCURE-0330 | RADCURE-0843 | RADCURE-1621 | RADCURE-2290 | RADCURE-2732 | RADCURE-3152 | RADCURE-3507 | RADCURE-3882 |
| RADCURE-0334 | RADCURE-0848 | RADCURE-1622 | RADCURE-2296 | RADCURE-2738 | RADCURE-3153 | RADCURE-3509 | RADCURE-3883 |
| RADCURE-0335 | RADCURE-0849 | RADCURE-1625 | RADCURE-2299 | RADCURE-2739 | RADCURE-3154 | RADCURE-3510 | RADCURE-3889 |
| RADCURE-0342 | RADCURE-0852 | RADCURE-1630 | RADCURE-2300 | RADCURE-2741 | RADCURE-3156 | RADCURE-3511 | RADCURE-3891 |
| RADCURE-0345 | RADCURE-0857 | RADCURE-1632 | RADCURE-2301 | RADCURE-2744 | RADCURE-3159 | RADCURE-3512 | RADCURE-3895 |
| RADCURE-0349 | RADCURE-0858 | RADCURE-1633 | RADCURE-2313 | RADCURE-2746 | RADCURE-3164 | RADCURE-3513 | RADCURE-3896 |
| RADCURE-0352 | RADCURE-0860 | RADCURE-1636 | RADCURE-2320 | RADCURE-2747 | RADCURE-3169 | RADCURE-3515 | RADCURE-3899 |
| RADCURE-0357 | RADCURE-0862 | RADCURE-1639 | RADCURE-2322 | RADCURE-2749 | RADCURE-3173 | RADCURE-3516 | RADCURE-3901 |
| RADCURE-0360 | RADCURE-0865 | RADCURE-1640 | RADCURE-2333 | RADCURE-2750 | RADCURE-3174 | RADCURE-3517 | RADCURE-3906 |
| RADCURE-0361 | RADCURE-0868 | RADCURE-1643 | RADCURE-2334 | RADCURE-2752 | RADCURE-3175 | RADCURE-3519 | RADCURE-3910 |
| RADCURE-0362 | RADCURE-0875 | RADCURE-1644 | RADCURE-2335 | RADCURE-2760 | RADCURE-3177 | RADCURE-3520 | RADCURE-3914 |
| RADCURE-0365 | RADCURE-0879 | RADCURE-1661 | RADCURE-2336 | RADCURE-2761 | RADCURE-3179 | RADCURE-3523 | RADCURE-3915 |
| RADCURE-0379 | RADCURE-0883 | RADCURE-1665 | RADCURE-2344 | RADCURE-2767 | RADCURE-3181 | RADCURE-3524 | RADCURE-3929 |
| RADCURE-0384 | RADCURE-0894 | RADCURE-1668 | RADCURE-2350 | RADCURE-2772 | RADCURE-3182 | RADCURE-3525 | RADCURE-3931 |
| RADCURE-0385 | RADCURE-0895 | RADCURE-1670 | RADCURE-2351 | RADCURE-2773 | RADCURE-3184 | RADCURE-3527 | RADCURE-3936 |
| RADCURE-0389 | RADCURE-0900 | RADCURE-1675 | RADCURE-2352 | RADCURE-2774 | RADCURE-3189 | RADCURE-3529 | RADCURE-3942 |
| RADCURE-0392 | RADCURE-0905 | RADCURE-1680 | RADCURE-2353 | RADCURE-2776 | RADCURE-3193 | RADCURE-3530 | RADCURE-3962 |
| RADCURE-0395 | RADCURE-0906 | RADCURE-1682 | RADCURE-2355 | RADCURE-2777 | RADCURE-3196 | RADCURE-3531 | RADCURE-3969 |
| RADCURE-0402 | RADCURE-0908 | RADCURE-1684 | RADCURE-2365 | RADCURE-2779 | RADCURE-3197 | RADCURE-3533 | RADCURE-3974 |
| RADCURE-0403 | RADCURE-0910 | RADCURE-1685 | RADCURE-2366 | RADCURE-2781 | RADCURE-3198 | RADCURE-3535 | RADCURE-3980 |
| RADCURE-0407 | RADCURE-0911 | RADCURE-1692 | RADCURE-2367 | RADCURE-2782 | RADCURE-3201 | RADCURE-3538 | RADCURE-3991 |
| RADCURE-0409 | RADCURE-0913 | RADCURE-1693 | RADCURE-2368 | RADCURE-2783 | RADCURE-3206 | RADCURE-3543 | RADCURE-3995 |
| RADCURE-0410 | RADCURE-0916 | RADCURE-1694 | RADCURE-2369 | RADCURE-2785 | RADCURE-3210 | RADCURE-3544 | RADCURE-4017 |
| RADCURE-0411 | RADCURE-0918 | RADCURE-1697 | RADCURE-2374 | RADCURE-2788 | RADCURE-3212 | RADCURE-3545 | RADCURE-4018 |
| RADCURE-0412 | RADCURE-0920 | RADCURE-1699 | RADCURE-2376 | RADCURE-2793 | RADCURE-3215 | RADCURE-3548 | RADCURE-4023 |
| RADCURE-0414 | RADCURE-0922 | RADCURE-1701 | RADCURE-2378 | RADCURE-2795 | RADCURE-3216 | RADCURE-3549 | RADCURE-4027 |
| RADCURE-0416 | RADCURE-0927 | RADCURE-1704 | RADCURE-2383 | RADCURE-2798 | RADCURE-3217 | RADCURE-3550 | RADCURE-4029 |
| RADCURE-0421 | RADCURE-0928 | RADCURE-1708 | RADCURE-2386 | RADCURE-2799 | RADCURE-3219 | RADCURE-3551 | RADCURE-4035 |
| RADCURE-0428 | RADCURE-0929 | RADCURE-1709 | RADCURE-2389 | RADCURE-2800 | RADCURE-3222 | RADCURE-3552 | RADCURE-4040 |
| RADCURE-0432 | RADCURE-0933 | RADCURE-1711 | RADCURE-2390 | RADCURE-2802 | RADCURE-3225 | RADCURE-3556 | RADCURE-4046 |
| RADCURE-0433 | RADCURE-0934 | RADCURE-1716 | RADCURE-2392 | RADCURE-2804 | RADCURE-3227 | RADCURE-3557 | RADCURE-4049 |
| RADCURE-0435 | RADCURE-0937 | RADCURE-1717 | RADCURE-2393 | RADCURE-2805 | RADCURE-3228 | RADCURE-3558 | RADCURE-4051 |
| RADCURE-0436 | RADCURE-0938 | RADCURE-1725 | RADCURE-2396 | RADCURE-2806 | RADCURE-3230 | RADCURE-3560 | RADCURE-4055 |
| RADCURE-0437 | RADCURE-0943 | RADCURE-1737 | RADCURE-2397 | RADCURE-2807 | RADCURE-3231 | RADCURE-3561 | RADCURE-4057 |
| RADCURE-0441 | RADCURE-0945 | RADCURE-1742 | RADCURE-2399 | RADCURE-2808 | RADCURE-3234 | RADCURE-3562 | RADCURE-4059 |
| RADCURE-0443 | RADCURE-0949 | RADCURE-1743 | RADCURE-2400 | RADCURE-2810 | RADCURE-3238 | RADCURE-3563 | RADCURE-4063 |
| RADCURE-0445 | RADCURE-0950 | RADCURE-1744 | RADCURE-2402 | RADCURE-2811 | RADCURE-3242 | RADCURE-3568 | RADCURE-4068 |
| RADCURE-0446 | RADCURE-0951 | RADCURE-1745 | RADCURE-2403 | RADCURE-2813 | RADCURE-3243 | RADCURE-3569 | RADCURE-4069 |
| RADCURE-0451 | RADCURE-0952 | RADCURE-1746 | RADCURE-2405 | RADCURE-2815 | RADCURE-3244 | RADCURE-3574 | RADCURE-4070 |
| RADCURE-0453 | RADCURE-0955 | RADCURE-1747 | RADCURE-2406 | RADCURE-2818 | RADCURE-3250 | RADCURE-3576 | RADCURE-4074 |
| RADCURE-0454 | RADCURE-0957 | RADCURE-1748 | RADCURE-2411 | RADCURE-2820 | RADCURE-3251 | RADCURE-3577 | RADCURE-4076 |
| RADCURE-0455 | RADCURE-0960 | RADCURE-1750 | RADCURE-2412 | RADCURE-2823 | RADCURE-3253 | RADCURE-3580 | RADCURE-4077 |
| RADCURE-0456 | RADCURE-0965 | RADCURE-1753 | RADCURE-2416 | RADCURE-2827 | RADCURE-3255 | RADCURE-3581 | RADCURE-4080 |
| RADCURE-0459 | RADCURE-0977 | RADCURE-1755 | RADCURE-2421 | RADCURE-2828 | RADCURE-3256 | RADCURE-3586 | RADCURE-4082 |
| RADCURE-0461 | RADCURE-0978 | RADCURE-1758 | RADCURE-2422 | RADCURE-2830 | RADCURE-3257 | RADCURE-3589 | RADCURE-4083 |
| RADCURE-0463 | RADCURE-0979 | RADCURE-1761 | RADCURE-2427 | RADCURE-2831 | RADCURE-3258 | RADCURE-3590 | RADCURE-4085 |
| RADCURE-0465 | RADCURE-0980 | RADCURE-1766 | RADCURE-2428 | RADCURE-2833 | RADCURE-3260 | RADCURE-3596 | RADCURE-4088 |
| RADCURE-0467 | RADCURE-0983 | RADCURE-1781 | RADCURE-2430 | RADCURE-2834 | RADCURE-3262 | RADCURE-3599 | RADCURE-4089 |
| RADCURE-0468 | RADCURE-0984 | RADCURE-1784 | RADCURE-2431 | RADCURE-2835 | RADCURE-3263 | RADCURE-3600 | RADCURE-4092 |
| RADCURE-0471 | RADCURE-0986 | RADCURE-1791 | RADCURE-2434 | RADCURE-2836 | RADCURE-3264 | RADCURE-3602 | RADCURE-4097 |
| RADCURE-0473 | RADCURE-0989 | RADCURE-1797 | RADCURE-2438 | RADCURE-2838 | RADCURE-3267 | RADCURE-3603 | RADCURE-4100 |
| RADCURE-0476 | RADCURE-0992 | RADCURE-1801 | RADCURE-2441 | RADCURE-2839 | RADCURE-3270 | RADCURE-3605 | RADCURE-4102 |
| RADCURE-0480 | RADCURE-0995 | RADCURE-1806 | RADCURE-2442 | RADCURE-2841 | RADCURE-3271 | RADCURE-3606 | RADCURE-4105 |
| RADCURE-0484 | RADCURE-0996 | RADCURE-1816 | RADCURE-2444 | RADCURE-2844 | RADCURE-3272 | RADCURE-3609 | RADCURE-4107 |
| RADCURE-0489 | RADCURE-1001 | RADCURE-1818 | RADCURE-2447 | RADCURE-2846 | RADCURE-3273 | RADCURE-3610 | RADCURE-4108 |
| RADCURE-0491 | RADCURE-1005 | RADCURE-1819 | RADCURE-2448 | RADCURE-2848 | RADCURE-3274 | RADCURE-3614 | RADCURE-4113 |
| RADCURE-0493 | RADCURE-1008 | RADCURE-1820 | RADCURE-2451 | RADCURE-2852 | RADCURE-3278 | RADCURE-3616 | RADCURE-4123 |
| RADCURE-0497 | RADCURE-1012 | RADCURE-1826 | RADCURE-2456 | RADCURE-2855 | RADCURE-3280 | RADCURE-3620 | RADCURE-4124 |
| RADCURE-0500 | RADCURE-1016 | RADCURE-1833 | RADCURE-2457 | RADCURE-2859 | RADCURE-3281 | RADCURE-3622 | RADCURE-4126 |
| RADCURE-0502 | RADCURE-1017 | RADCURE-1837 | RADCURE-2459 | RADCURE-2862 | RADCURE-3289 | RADCURE-3624 | RADCURE-4127 |
| RADCURE-0505 | RADCURE-1018 | RADCURE-1842 | RADCURE-2465 | RADCURE-2863 | RADCURE-3291 | RADCURE-3625 | RADCURE-4129 |
| RADCURE-0510 | RADCURE-1020 | RADCURE-1843 | RADCURE-2468 | RADCURE-2864 | RADCURE-3293 | RADCURE-3626 | RADCURE-3631 |
| RADCURE-0512 | RADCURE-1022 | RADCURE-1848 | RADCURE-2469 | RADCURE-2866 | RADCURE-3294 | RADCURE-3628 | RADCURE-3632 |
| RADCURE-0513 | RADCURE-1024 | RADCURE-1850 | RADCURE-2470 | RADCURE-2867 | RADCURE-3295 | RADCURE-3629 | RADCURE-3634 |

Table S2. index of patients eligible for inclusion in the H&N1 dataset

| HN1004 | HN1088 | HN1201 | HN1339 | HN1429 | HN1560 | HN1813 | HN1933 |
| --- | --- | --- | --- | --- | --- | --- | --- |
| HN1006 | HN1092 | HN1215 | HN1344 | HN1469 | HN1572 | HN1827 | HN1954 |
| HN1022 | HN1096 | HN1244 | HN1356 | HN1483 | HN1609 | HN1838 | HN1987 |
| HN1029 | HN1102 | HN1259 | HN1367 | HN1487 | HN1640 | HN1839 |  |
| HN1046 | HN1117 | HN1260 | HN1368 | HN1488 | HN1648 | HN1851 |  |
| HN1054 | HN1135 | HN1263 | HN1369 | HN1491 | HN1703 | HN1869 |  |
| HN1060 | HN1146 | HN1271 | HN1372 | HN1501 | HN1719 | HN1879 |  |
| HN1067 | HN1180 | HN1294 | HN1395 | HN1517 | HN1748 | HN1900 |  |
| HN1079 | HN1192 | HN1305 | HN1400 | HN1519 | HN1791 | HN1901 |  |
| HN1080 | HN1197 | HN1319 | HN1412 | HN1549 | HN1792 | HN1910 |  |
| HN1081 | HN1200 | HN1323 | HN1417 | HN1555 | HN1805 | HN1913 |  |

Table S3. Radiomics features calculated by using Pyradiomics

| Index | Features | Index | Features |
| --- | --- | --- | --- |
| 1 | original_shape_Elongation | 53 | original_shape_Flatness |
| 2 | original_shape_LeastAxisLength | 54 | original_shape_MajorAxisLength |
| 3 | original_shape_Maximum2DDiameterColumn | 55 | original_shape_Maximum2DDiameterRow |
| 4 | original_shape_Maximum2DDiameterSlice | 56 | original_shape_MeshVolume |
| 5 | original_shape_Maximum3DDiameter | 57 | original_shape_MinorAxisLength |
| 6 | original_shape_Sphericity | 58 | original_shape_SurfaceArea |
| 7 | original_firstorder_10Percentile | 59 | original_glrlm_LongRunLowGrayLevelEmphasis |
| 8 | original_firstorder_90Percentile | 60 | original_glrlm_LowGrayLevelRunEmphasis |
| 9 | original_firstorder_Energy | 61 | original_glrlm_RunEntropy |
| 10 | original_firstorder_Entropy | 62 | original_glrlm_RunLengthNonUniformity |
| 11 | original_firstorder_InterquartileRange | 63 | original_glrlm_RunLengthNonUniformityNormalized |
| 12 | original_firstorder_Kurtosis | 64 | original_glrlm_RunPercentage |
| 13 | original_firstorder_Maximum | 65 | original_glrlm_RunVariance |
| 14 | original_firstorder_Mean | 66 | original_glrlm_ShortRunEmphasis |
| 15 | original_firstorder_MeanAbsoluteDeviation | 67 | original_glrlm_ShortRunHighGrayLevelEmphasis |
| 16 | original_firstorder_Median | 68 | original_glrlm_ShortRunLowGrayLevelEmphasis |
| 17 | original_firstorder_Minimum | 69 | original_glszm_GrayLevelNonUniformity |
| 18 | original_firstorder_Range | 70 | original_glszm_GrayLevelNonUniformityNormalized |
| 19 | original_firstorder_RobustMeanAbsoluteDeviation | 71 | original_glszm_GrayLevelVariance |
| 20 | original_firstorder_RootMeanSquared | 72 | original_glszm_HighGrayLevelZoneEmphasis |
| 21 | original_firstorder_Skewness | 73 | original_glszm_LargeAreaEmphasis |
| 22 | original_firstorder_Uniformity | 74 | original_glszm_LargeAreaHighGrayLevelEmphasis |
| 23 | original_firstorder_Variance | 75 | original_glszm_LargeAreaLowGrayLevelEmphasis |
| 24 | original_glcm_Autocorrelation | 76 | original_glszm_LowGrayLevelZoneEmphasis |
| 25 | original_glcm_JointAverage | 77 | original_glszm_SizeZoneNonUniformity |
| 26 | original_glcm_ClusterProminence | 78 | original_glszm_SizeZoneNonUniformityNormalized |
| 27 | original_glcm_ClusterShade | 79 | original_glszm_SmallAreaEmphasis |
| 28 | original_glcm_ClusterTendency | 80 | original_glszm_SmallAreaHighGrayLevelEmphasis |
| 29 | original_glcm_Contrast | 81 | original_glszm_SmallAreaLowGrayLevelEmphasis |
| 30 | original_glcm_Correlation | 82 | original_glszm_ZoneEntropy |
| 31 | original_glcm_DifferenceAverage | 83 | original_glszm_ZonePercentage |
| 32 | original_glcm_DifferenceEntropy | 84 | original_glszm_ZoneVariance |
| 33 | original_glcm_DifferenceVariance | 85 | original_gldm_DependenceEntropy |
| 34 | original_glcm_JointEnergy | 86 | original_gldm_DependenceNonUniformity |
| 35 | original_glcm_JointEntropy | 87 | original_gldm_DependenceNonUniformityNormalized |
| 36 | original_glcm_Imc1 | 88 | original_gldm_DependenceVariance |
| 37 | original_glcm_Imc2 | 89 | original_gldm_GrayLevelNonUniformity |
| 38 | original_glcm_Idm | 90 | original_gldm_GrayLevelVariance |
| 39 | original_glcm_Idmn | 91 | original_gldm_HighGrayLevelEmphasis |
| 40 | original_glcm_Id | 92 | original_gldm_LargeDependenceEmphasis |
| 41 | original_glcm_Idn | 93 | original_gldm_LargeDependenceHighGrayLevelEmphasis |
| 42 | original_glcm_InverseVariance | 94 | original_gldm_LargeDependenceLowGrayLevelEmphasis |
| 43 | original_glcm_MaximumProbability | 95 | original_gldm_LowGrayLevelEmphasis |
| 44 | original_glcm_SumEntropy | 96 | original_gldm_SmallDependenceEmphasis |
| 45 | original_glcm_SumSquares | 97 | original_gldm_SmallDependenceHighGrayLevelEmphasis |
| 46 | original_glrlm_GrayLevelNonUniformity | 98 | original_gldm_SmallDependenceLowGrayLevelEmphasis |
| 47 | original_glrlm_GrayLevelNonUniformityNormalized | 99 | original_ngtdm_Busyness |
| 48 | original_glrlm_GrayLevelVariance | 100 | original_ngtdm_Coarseness |
| 49 | original_glrlm_HighGrayLevelRunEmphasis | 101 | original_ngtdm_Complexity |
| 50 | original_glrlm_LongRunEmphasis | 102 | original_ngtdm_Contrast |
| 51 | original_glrlm_LongRunHighGrayLevelEmphasis | 103 | original_ngtdm_Strength |
| 52 | original_shape_SurfaceVolumeRatio |  |  |

Table S4. Parameters of params.yaml for radiomics extraction. In YAML, params.yaml refers to the parameters section that defines what parameters are available for a pipeline.

| Parameters | Value |
| --- | --- |
| binWidth | 25 |
| Interpolator | 'sitkBSpline' |
| resampledPixelSpacing | [2, 2, 2] |
| padDistance | 10 |
| resegmentRange | [-3, 3] |
| resegmentMode | sigma |
| voxelArrayShift | 1000 |
| Label | 1 |

Table S5. Label for Kinetics-400 Dataset

| Index | Label | Index | Label | Index | Label | Index |  |
| --- | --- | --- | --- | --- | --- | --- | --- |
| 0 | abseiling | 100 | drinking | 200 | moving furniture | 300 | shredding paper |
| 1 | air drumming | 101 | drinking beer | 201 | mowing lawn | 301 | shuffling cards |
| 2 | answering questions | 102 | drinking shots | 202 | news anchoring | 302 | side kick |
| 3 | applauding | 103 | driving car | 203 | opening bottle | 303 | sign language interpreting |
| 4 | applying cream | 104 | driving tractor | 204 | opening present | 304 | singing |
| 5 | archery | 105 | drop kicking | 205 | paragliding | 305 | situp |
| 6 | arm wrestling | 106 | drumming fingers | 206 | parasailing | 306 | skateboarding |
| 7 | arranging flowers | 107 | dunking basketball | 207 | parkour | 307 | ski jumping |
| 8 | assembling computer | 108 | dying hair | 208 | passing American football (in game) | 308 | skiing (not slalom or crosscountry) |
| 9 | auctioning | 109 | eating burger | 209 | passing American football (not in game) | 309 | skiing crosscountry |
| 10 | baby waking up | 110 | eating cake | 210 | peeling apples | 310 | skiing slalom |
| 11 | baking cookies | 111 | eating carrots | 211 | peeling potatoes | 311 | skipping rope |
| 12 | balloon blowing | 112 | eating chips | 212 | petting animal (not cat) | 312 | skydiving |
| 13 | bandaging | 113 | eating doughnuts | 213 | petting cat | 313 | slacklining |
| 14 | barbequing | 114 | eating hotdog | 214 | picking fruit | 314 | slapping |
| 15 | bartending | 115 | eating ice cream | 215 | planting trees | 315 | sled dog racing |
| 16 | beatboxing | 116 | eating spaghetti | 216 | plastering | 316 | smoking |
| 17 | bee keeping | 117 | eating watermelon | 217 | playing accordion | 317 | smoking hookah |
| 18 | belly dancing | 118 | egg hunting | 218 | playing badminton | 318 | snatch weight lifting |
| 19 | bench pressing | 119 | exercising arm | 219 | playing bagpipes | 319 | sneezing |
| 20 | bending back | 120 | exercising with an exercise ball | 220 | playing basketball | 320 | sniffing |
| 21 | bending metal | 121 | extinguishing fire | 221 | playing bass guitar | 321 | snorkeling |
| 22 | biking through snow | 122 | faceplanting | 222 | playing cards | 322 | snowboarding |
| 23 | blasting sand | 123 | feeding birds | 223 | playing cello | 323 | snowkiting |
| 24 | blowing glass | 124 | feeding fish | 224 | playing chess | 324 | snowmobiling |
| 25 | blowing leaves | 125 | feeding goats | 225 | playing clarinet | 325 | somersaulting |
| 26 | blowing nose | 126 | filling eyebrows | 226 | playing controller | 326 | spinning poi |
| 27 | blowing out candles | 127 | finger snapping | 227 | playing cricket | 327 | spray painting |
| 28 | bobsledding | 128 | fixing hair | 228 | playing cymbals | 328 | spraying |
| 29 | bookbinding | 129 | flipping pancake | 229 | playing didgeridoo | 329 | springboard diving |
| 30 | bouncing on trampoline | 130 | flying kite | 230 | playing drums | 330 | squat |
| 31 | bowling | 131 | folding clothes | 231 | playing flute | 331 | sticking tongue out |
| 32 | braiding hair | 132 | folding napkins | 232 | playing guitar | 332 | stomping grapes |
| 33 | breading or breadcrumbing | 133 | folding paper | 233 | playing harmonica | 333 | stretching arm |
| 34 | breakdancing | 134 | front raises | 234 | playing harp | 334 | stretching leg |
| 35 | brush painting | 135 | frying vegetables | 235 | playing ice hockey | 335 | strumming guitar |
| 36 | brushing hair | 136 | garbage collecting | 236 | playing keyboard | 336 | surfing crowd |
| 37 | brushing teeth | 137 | gargling | 237 | playing kickball | 337 | surfing water |
| 38 | building cabinet | 138 | getting a haircut | 238 | playing monopoly | 338 | sweeping floor |
| 39 | building shed | 139 | getting a tattoo | 239 | playing organ | 339 | swimming backstroke |
| 40 | bungee jumping | 140 | giving or receiving award | 240 | playing paintball | 340 | swimming breast stroke |
| 41 | busking | 141 | golf chipping | 241 | playing piano | 341 | swimming butterfly stroke |
| 42 | canoeing or kayaking | 142 | golf driving | 242 | playing poker | 342 | swing dancing |
| 43 | capoeira | 143 | golf putting | 243 | playing recorder | 343 | swinging legs |
| 44 | carrying baby | 144 | grinding meat | 244 | playing saxophone | 344 | swinging on something |
| 45 | cartwheeling | 145 | grooming dog | 245 | playing squash or racquetball | 345 | sword fighting |
| 46 | carving pumpkin | 146 | grooming horse | 246 | playing tennis | 346 | tai chi |
| 47 | catching fish | 147 | gymnastics tumbling | 247 | playing trombone | 347 | taking a shower |
| 48 | catching or throwing baseball | 148 | hammer throw | 248 | playing trumpet | 348 | tango dancing |
| 49 | catching or throwing frisbee | 149 | headbanging | 249 | playing ukulele | 349 | tap dancing |
| 50 | catching or throwing softball | 150 | headbutting | 250 | playing violin | 350 | tapping guitar |
| 51 | celebrating | 151 | high jump | 251 | playing volleyball | 351 | tapping pen |
| 52 | changing oil | 152 | high kick | 252 | playing xylophone | 352 | tasting beer |
| 53 | changing wheel | 153 | hitting baseball | 253 | pole vault | 353 | tasting food |
| 54 | checking tires | 154 | hockey stop | 254 | presenting weather forecast | 354 | testifying |
| 55 | cheerleading | 155 | holding snake | 255 | pull ups | 355 | texting |
| 56 | chopping wood | 156 | hopscotch | 256 | pumping fist | 356 | throwing axe |
| 57 | clapping | 157 | hoverboarding | 257 | pumping gas | 357 | throwing ball |
| 58 | clay pottery making | 158 | hugging | 258 | punching bag | 358 | throwing discus |
| 59 | clean and jerk | 159 | hula hooping | 259 | punching person (boxing) | 359 | tickling |
| 60 | cleaning floor | 160 | hurdling | 260 | push up | 360 | tobogganing |
| 61 | cleaning gutters | 161 | hurling (sport) | 261 | pushing car | 361 | tossing coin |
| 62 | cleaning pool | 162 | ice climbing | 262 | pushing cart | 362 | tossing salad |
| 63 | cleaning shoes | 163 | ice fishing | 263 | pushing wheelchair | 363 | training dog |
| 64 | cleaning toilet | 164 | ice skating | 264 | reading book | 364 | trapezing |
| 65 | cleaning windows | 165 | ironing | 265 | reading newspaper | 365 | trimming or shaving beard |
| 66 | climbing a rope | 166 | javelin throw | 266 | recording music | 366 | trimming trees |
| 67 | climbing ladder | 167 | jetskiing | 267 | riding a bike | 367 | triple jump |
| 68 | climbing tree | 168 | jogging | 268 | riding camel | 368 | tying bow tie |
| 69 | contact juggling | 169 | juggling balls | 269 | riding elephant | 369 | tying knot (not on a tie) |
| 70 | cooking chicken | 170 | juggling fire | 270 | riding mechanical bull | 370 | tying tie |
| 71 | cooking egg | 171 | juggling soccer ball | 271 | riding mountain bike | 371 | unboxing |
| 72 | cooking on campfire | 172 | jumping into pool | 272 | riding mule | 372 | unloading truck |
| 73 | cooking sausages | 173 | jumpstyle dancing | 273 | riding or walking with horse | 373 | using computer |
| 74 | counting money | 174 | kicking field goal | 274 | riding scooter | 374 | using remote controller (not gaming) |
| 75 | country line dancing | 175 | kicking soccer ball | 275 | riding unicycle | 375 | using segway |
| 76 | cracking neck | 176 | kissing | 276 | ripping paper | 376 | vault |
| 77 | crawling baby | 177 | kitesurfing | 277 | robot dancing | 377 | waiting in line |
| 78 | crossing river | 178 | knitting | 278 | rock climbing | 378 | walking the dog |
| 79 | crying | 179 | krumping | 279 | rock scissors paper | 379 | washing dishes |
| 80 | curling hair | 180 | laughing | 280 | roller skating | 380 | washing feet |
| 81 | cutting nails | 181 | laying bricks | 281 | running on treadmill | 381 | washing hair |
| 82 | cutting pineapple | 182 | long jump | 282 | sailing | 382 | washing hands |
| 83 | cutting watermelon | 183 | lunge | 283 | salsa dancing | 383 | water skiing |
| 84 | dancing ballet | 184 | making a cake | 284 | sanding floor | 384 | water sliding |
| 85 | dancing charleston | 185 | making a sandwich | 285 | scrambling eggs | 385 | watering plants |
| 86 | dancing gangnam style | 186 | making bed | 286 | scuba diving | 386 | waxing back |
| 87 | dancing macarena | 187 | making jewelry | 287 | setting table | 387 | waxing chest |
| 88 | deadlifting | 188 | making pizza | 288 | shaking hands | 388 | waxing eyebrows |
| 89 | decorating the christmas tree | 189 | making snowman | 289 | shaking head | 389 | waxing legs |
| 90 | digging | 190 | making sushi | 290 | sharpening knives | 390 | weaving basket |
| 91 | dining | 191 | making tea | 291 | sharpening pencil | 391 | welding |
| 92 | disc golfing | 192 | marching | 292 | shaving head | 392 | whistling |
| 93 | diving cliff | 193 | massaging back | 293 | shaving legs | 393 | windsurfing |
| 94 | dodgeball | 194 | massaging feet | 294 | shearing sheep | 394 | wrapping present |
| 95 | doing aerobics | 195 | massaging legs | 295 | shining shoes | 395 | wrestling |
| 96 | doing laundry | 196 | massaging person's head | 296 | shooting basketball | 396 | writing |
| 97 | doing nails | 197 | milking cow | 297 | shooting goal (soccer) | 397 | yawning |
| 98 | drawing | 198 | mopping floor | 298 | shot put | 398 | yoga |
| 99 | dribbling basketball | 199 | motorcycling | 299 | shoveling snow | 399 | zumba |
